# BODIPY-Labeled Estrogens for Fluorescence Analysis of Environmental Microbial Degradation

**DOI:** 10.1021/acsomega.2c05002

**Published:** 2022-11-03

**Authors:** Celeste Felion, Ricardo Lopez-Gonzalez, Alan L. Sewell, Rodolfo Marquez, Caroline Gauchotte-Lindsay

**Affiliations:** † James Watt School of Engineering, University of Glasgow, GlasgowG12 6EW, U.K.; ‡ School of Chemistry, University of Glasgow, GlasgowG12 8QQ, U.K.; § School of Physical and Chemical Sciences, University of Canterbury, Christchurch8140, New Zealand

## Abstract

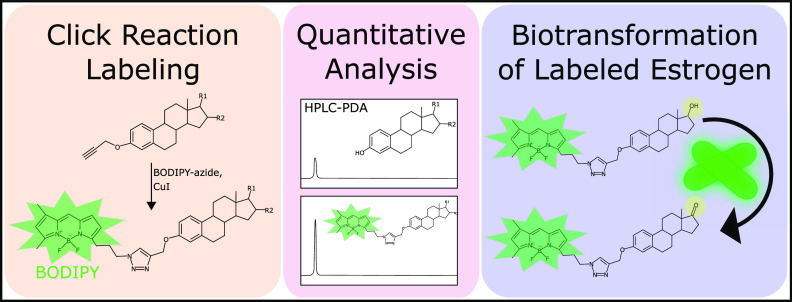

Biodegradation of estrogen hormone micropollutants is a well-established approach toward their remediation. Fluorescently labeled substrates are used extensively for rapid, near-real-time analysis of biological processes and are a potential tool for studying biodegradation processes faster and more efficiently than conventional approaches. However, it is important to understand how the fluorescently tagged surrogates compare with the natural substrate in terms of chemical analysis and the intended application. We derivatized three natural estrogens with BODIPY fluorophores by azide–alkyne cycloaddition click reaction and developed an analytical workflow based on simple liquid–liquid extraction and HPLC-PDA analysis. The developed methods allow for concurrent analysis of both fluorescent and natural estrogens with comparable recovery, accuracy, and precision. We then evaluated the use of BODIPY-labeled estrogens as surrogate substrates for studying biodegradation using a model bacterium for estrogen metabolism. The developed analytical methods were successfully employed to compare the biological transformation of 17β-estradiol (E2), with and without the BODIPY fluorescent tag. Through measuring the complete degradation of E2 and the transformation of BODIPY-estradiol to BODIPY-estrone in the presence of a co-substrate, we found that BODIPY-labeled estrogens are biologically viable surrogates for investigating biodegradation in environmental bacteria.

## Introduction

Estrogens are steroidal hormones which have been designated as endocrine-disrupting chemicals.^1^ The deleterious effects of natural and synthetic estrogens on aquatic ecology has been repeatedly demonstrated for nearly 25 years, particularly through production of the female-associated protein vitellogenin in male oviparous animals.^1−3^ As a result, environmental authorities across the world have included the most prevalent estrogens—estrone (E1), 17β-estradiol (E2), estriol (E3), and synthetic estrogen 17α-ethinylestradiol (EE2)—in new monitoring watch lists of emerging contaminants in water systems.^4,5^

The role of biological degradation in removing estrogen hormones from water treatment systems has been reported in numerous studies, including aerobic degradation through activated sludge^6−8^ and biofilters^9^ and anaerobic degradation in waste lagoons.^10,11^ Recently, the specific biodegradation pathways of estrogen-degrading environmental bacteria have been elucidated, including *Nitrosomonas europaea*,^12,13^*Novosphingobium* spp.,^14^*Sphingomonas* spp.,^15,16^*Sphingobacterium* spp.,^17^ and *Novosphingobium tardaugens*.^18^ However, identifying, isolating, and studying estrogen-degrading bacteria is often a time- and labor-intensive process, with lengthy enrichment culture periods ranging from several weeks up to a year, time-consuming sample preparation, and low-throughput chromatographic analyses.^19−21^ Furthermore, in contrast with biomedical research, isolating bacteria from the environment is extremely challenging as most microorganisms are unculturable in lab settings.^22^

There has been increasing interest in utilizing fluorescently labeled substrates to study biochemical processes in near real time. Fluorescent probes are widely used in fluorescence microscopy for spatial in situ analysis^23,24^ and as sensors for biochemical assays.^25−27^ A recent publication by Leivers et al. (2022) demonstrated the use of 2-aminobenzamide fluorophore in a multi-technique approach to study substrate-specific biodegradation of glycans by gut microbiome, highlighting the breadth of information that can be gleaned using fluorescent probes.^28^ Although most applications have been in biomedical research, fluorescent probes have also been used in environmental science, for example, to study the distributions of Giardia cysts^29^ and heavy metals in microbial communities.^30^ Advances in organic fluorophores and conjugation techniques have expanded the possibilities for research using fluorescent probes.^31^ In particular, 4,4-difluoro-4-bora-3a,4a-diaza-*s*-indacene (BODIPY) dyes are used extensively in bioconjugation due to their excellent photophysical properties, such as intense fluorescence, high degree of photostability, and a scaffold that is readily tunable to different excitation and emission wavelengths.^32−34^

Although BODIPY fluorophores possess several advantages, there are some practical challenges in using them in biological research. BODIPY dyes are widely known to form dimers and aggregates in polar solutions which affect their solubility, bioavailability, and fluorescence intensity.^35,36^ Traditionally, water solubility has been improved by derivatizing the BODIPY core with hydrophilic groups, including galactose,^37^ sulfonate, phosphonate, and carboxylate groups;^38^ however, these groups increase the steric hindrance and reduce the chemical stability of the fluorophore. Furthermore, for small molecules, the fluorophore drastically increases the molecular weight and changes the chemical nature of the parent substrate. This presents a twofold challenge in using BODIPY probes in biological systems: the poor aqueous solubility and the impact of conjugated fluorophore on metabolism. Thus, before confidently using BODIPY conjugates of micropollutants to investigate biodegrading microorganisms, it is important to understand the impact of BODIPY conjugation on the solubility of the substrate and on its biological activity using well-characterized reference strains. Chromatographic analyses of the substrate products are crucial for validating fluorescently tagged substrates as viable surrogates.^28,39^ However, presently, there is limited information about the analytical methods used to extract and analyze BODIPY-conjugated molecules compared to their native structure.

**Figure 1:**
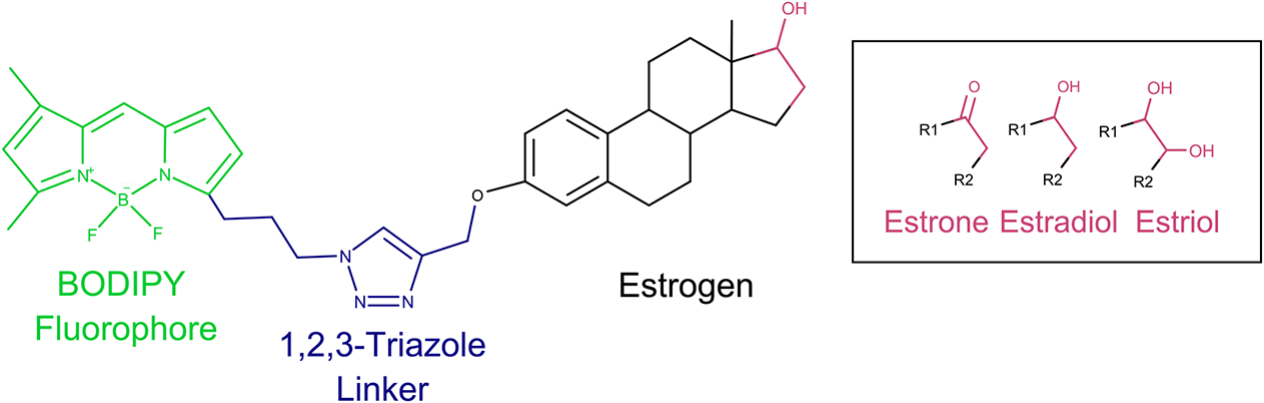
Structures of BODIPY-conjugated estrogens used in this work. The BODIPY fluorophore was conjugated to estrone, 17β-estradiol, and estriol via an aromatic triazole linker ligated to the oxygen at C3 of the aromatic A-ring of estrogen.

In this study, we compared the biodegradation of natural estrogens and estrogens derivatized with BODIPY by the estrogen-catabolizing bacterium, *Caenibius tardaugens* strain DSM 16702 (formerly identified as *N. tardaugens* ARI-1), which was originally isolated from the activated sludge of a Tokyo sewage treatment plant by Fujii et al. (2003).^40^ First, dimethyl azido-BODIPY fluorophore was successfully synthesized and conjugated to the primary natural estrogens (Figure 1 and Table 1) via a Cu(I)-catalyzed cycloaddition “click” reaction,^34^ followed by addition of a terminal alkyne to the C3 hydroxyl group. Next, we developed a robust analytical workflow which accounts for the insoluble and aggregative properties of BODIPY, without the addition of hydrophilic functional groups. Finally, we used the developed analytical method to quantitatively investigate how *C. tardaugens* metabolizes our synthesized BODIPY-estradiol compared to the native structure.

**Table 1:** Relevant Physical Properties of the Estrogens Used in This Study^a^

Analyte	ID	Analysis	Abs λ_max_ (nm)	Formula	Molecular Weight (Da)
Estrone	E1	HPLC@230 nm	280	C_18_H_22_O_2_	270.37
17β-Estradiol	E2	HPLC@230 nm	280	C_18_H_24_O_2_	272.38
Estriol	E3	HPLC@230 nm	280	C_18_H_24_O_3_	288.39
BODIPY-Estrone	BDP-E1	HPLC@503 nm	503	C_35_H_40_O_2_N_5_BF_2_	611.53
BODIPY-17β-Estradiol	BDP-E2	HPLC@503 nm	503	C_35_H_42_O_2_N_5_BF_2_	613.55
BODIPY-Estriol	BDP-E3	HPLC@503 nm	503	C_35_H_42_O_3_N_5_BF_2_	629.55
BODIPY-Azide	BDP-N3	HPLC@503 nm	503	C_14_H_16_BF_2_N_5_	303.12

a BODIPY-azide represents the BODIPY fluorophore (compound **X5** in the Supporting Information. I—Synthesis of BODIPY and BODIPY-Linked Estrogens).

## Results and Discussion

### Synthesis of BODIPY-Estrogen Conjugates

Previously published methods for azido-BODIPY fluorophore synthesis and conjugation with terminal alkyne via the copper-catalyzed click reaction were used here for producing BODIPY-estrogen conjugates.^34^ The BODIPY fluorophore used in this work is very minimally substituted and contains no hydrophilic functional groups to enhance solubility (Figure 1). This decision serves two purposes. First, this adds the least possible molecular surface area to the conjugated estrogen to minimize steric and functional interference in metabolic activity. Second, we are able to explore the fate of the BODIPY core structure in biological experiments.

**Scheme 1:**
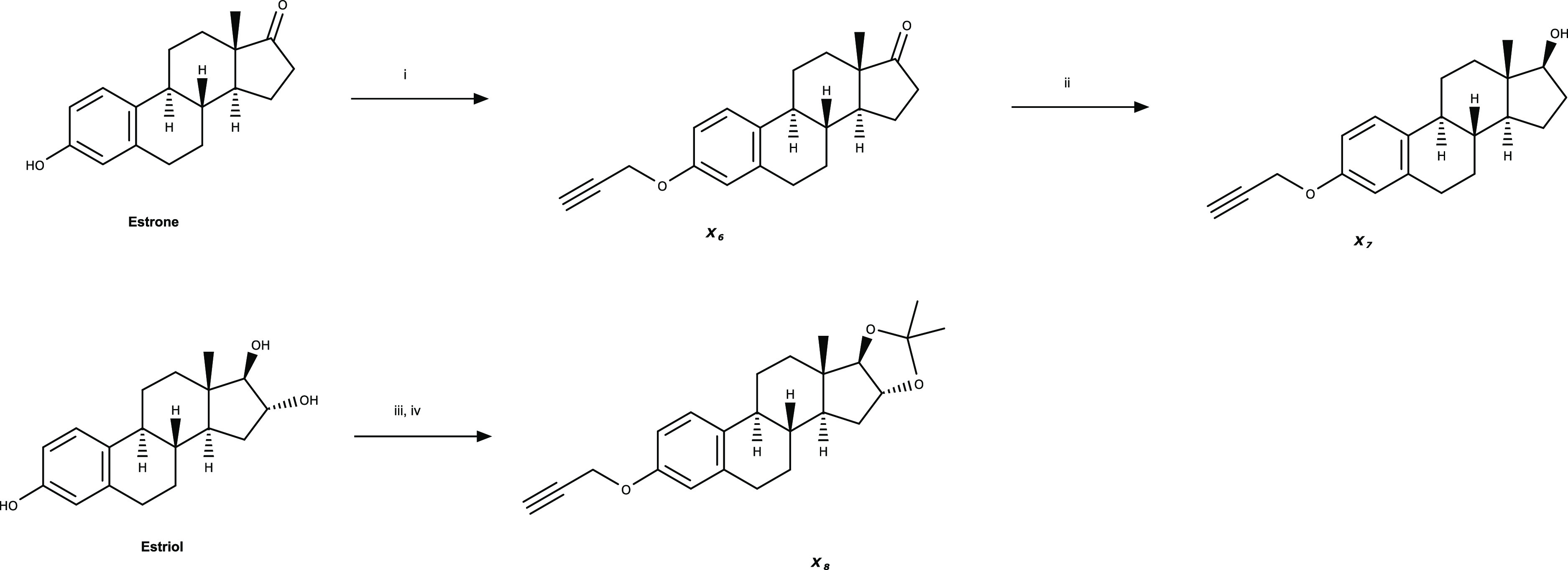
Synthesis of Alkylated Estrone (**X6**), 17β-Estradiol (**X7**), and Estriol (**X8**) Reagents and conditions: (i) propargyl bromide, K_2_CO_3_, DMF, 70 °C, 16 h, and 86%; (ii) NaBH_4_, aq. MeOH/CH_2_Cl_2_, rt, 2 h, and 86%; (iii) 2-methoxypropene, *p*-TsOH, acetone/THF, 0 °C to rt, and 4 h; and (iv) propargyl bromide, K_2_CO_3_, PhMe/DMF, 70 °C, 16 h, and 60% over two steps.

Initially, estrone was reacted with bromobut-3-yne under a variety of conditions without any conversion to the expected product. The reaction was then attempted with propargyl bromide, which provides a shorter link between the hormone and fluorescent core, to yield the desired 3-*O*-propargylestone (**X6**) in good yield. In order to circumvent the failure to alkylate 17β-estradiol, using the same conditions, it was decided to reduce the alkylated estrone (**X6**) to afford the expected 3-*O*-propargyl-β-estradiol (**X7**) in good yield. On the other hand, 1,2-diol of estriol was ketal-protected prior to attempting alkylation to avoid the uncontrolled alkylation observed with 17β-estradiol. The crude product was then treated with propargyl bromide to yield 3-*O*-propargyl-(16-*O*,17-*O*-dimethylacetyl)estriol (**X8**) in moderated yield after two steps (Scheme 1).

**Scheme 2:**
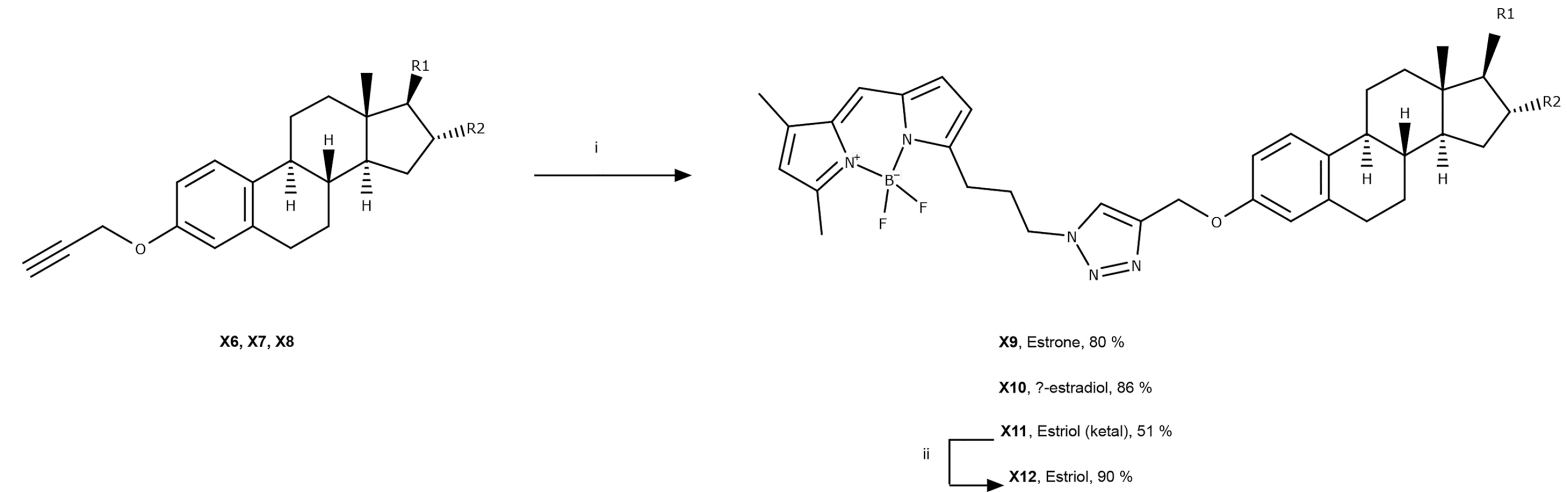
Synthesis of BODIPY-Tagged Estrogens (**X9**–**X12**) Reagents and conditions: (i) azido-BODIPY, CuI, DIPEA, THF, 40 °C, 16 h, and 51–86% and (ii) BiCl_3_, aq MeCN, rt, 3 h, and 90%.

Having on hand the alkylated estrogens, the azido-BODIPY block was successfully synthesized following a developed route into the group. Finally, the coupling of alkylated primary natural estrogens with azido-BODIPY via the Cu (I)-catalyzed cycloaddition “click” reaction afforded the expected fluorescent derivatives: estrone (**X9**), 17β-estradiol (**X10**), and estriol (ketal) (**X11**) in moderate to high yield. Subsequent deprotection in mild acidic conditions of the ketal-protected analogue **X11** afforded the desired fluorescent estriol derivative (**X12**) in high yield (Scheme 2).

The NMR results for the synthesized BODIPY tag, BODIPY-azide, closely matched that of previous results (Table 2).^34^ In addition, the measured mass-to-charge ratios for the BODIPY-estrogens very closely match the predicted values for the sodium adduct ions. The full results for each synthesis intermediate can be found in Supporting Information I—Synthesis of BODIPY and BODIPY-Linked Estrogens. The NMR and mass spectrometry spectra for the three BODIPY-tagged estrogens (**X9**, **X10**, and **X12**) are provided in Supporting Information VI—BODIPY-Estrogens NMR and HRMS Spectra.

**Table 2:** NMR and High-Resolution Mass Spectrometry (HRMS) Data for Synthesized BODIPY-Azide (N3) and BODIPY-Estrogens

Analyte	Analysis	Results
BODIPY-N3, **X5**	^1^H NMR δ	7.11 (1H, s), 6.94 (1H, d, *J* = 4.0 Hz), 6.31 (1H, d, *J* = 4.0 Hz), 6.14 (1H, s), 3.41 (2H, t, *J* = 8.0 Hz), 3.07 (2H, t, *J* = 8.0 Hz), 2.59 (3H, s), 2.28 (3H, s), 2.06–2.01 (2H, m)
	^13^C NMR δ	160.5, 156.5, 146.7, 143.7, 134.1, 128.3, 123.8, 120.2, 116.6, 50.9, 28.2, 25.8, 14.9, 11.4
BODIPY-E1, **X9**	^1^H NMR δ	7.63 (1H, s), 7.20 (1H, d, *J* = 8.5 Hz), 7.09 (1H, s), 6.88 (1H, d, *J* = 4.0 Hz), 6.79 (1H, dd, *J* = 8.5, 2.7 Hz), 6.73 (1H, d, *J* = 2.7 Hz), 6.24 (1H, d, *J* = 4.0 Hz), 6.13 (1H, s), 5.18 (2H, s), 4.43 (2H, t, *J* = 7.3 Hz), 3.04 (2H, t, *J* = 7.3 Hz), 2.91–2.87 (2H, m), 2.57 (3H, s), 2.53–2.47 (1H, m), 2.42–2.36 (3H, m), 2.25 (3H, s), 2.25–1.94 (5H, m), 1.71–1.41 (6H, m), 0.91 (3H, s)
	^13^C NMR δ	220.6, 160.3, 156.6,156.3, 144.4, 144.1, 137.9, 135.3, 133.2, 132.6, 128.2, 126.4, 123.9, 122.8, 120.6, 116.7, 114.8, 112.4, 62.1, 50.4, 49.8, 48.0, 44.0, 38.3, 35.9, 31.6, 29.6, 29.5, 26.5, 25.9, 25.7, 21.6, 15.0, 13.9, 11.3
	HRMS	calcd for C_35_H_40_F_2_N_5_NaO_2_B [M + Na]^+^: *m/z* 633.3172. Found *m/z*: 633.3162
BODIPY-E2, **X10**	^1^H NMR δ	7.20 (1H, d, *J* = 8.6 Hz), 7.09 (1H, s), 6.88 (1H, d, *J* = 3.9 Hz), 6.78 (1H, dd, *J* = 8.6, 2.7 Hz), 6.71 (1H, d, *J* = 2.7 Hz), 6.24 (1H, d, *J* = 3.9 Hz),6.13 (1H, s), 5.17 (2H, s), 4.43 (2H, t, *J* = 7.3 Hz), 3.73 (1H, t, *J* = 8.6 Hz), 3.04 (2H, t, *J* = 7.3 Hz), 2.87–2.83 (2H, m), 2.57 (3H, s), 2.42–2.36 (1H, m), 2.32–2.08 (3H, m), 2.25 (3H, s), 1.97–1.93 (1H, m),1.90–1.85 (1H, m), 1.73–1.67 (1H, m), 1.52–1.16, (9H, m), 0.78 (3H, s)
	^13^C NMR δ	160.6, 156.6, 156.2, 144.4, 144.1, 138.1,135.4, 133.2, 133.5, 128.2, 126.4, 123.8, 122.8, 120.5, 116.7, 114.8, 112.3, 81.9, 62.1, 50.1, 49.8, 44.0, 43.3, 38.8, 36.7, 30.6, 29.8, 29.5, 27.2, 26.3, 25.7, 23.1, 15.0, 11.3, 11.1
	HRMS	calcd for C_35_H_42_F_2_N_5_NaO_3_B [M + Na]^+^: *m/z* 633.3172. Found *m/z*: 633.3162
BODIPY-E3, **X12**	^1^H NMR δ	7.64 (1H, s), 7.18 (1H, d, *J* = 8.6 Hz), 7.09 (1H, s), 6.88 (1H, d, *J* = 3.9 Hz), 6.77 (1H, dd, *J* = 8.6, 2.6 Hz), 6.70 (1H, d, *J* = 2.6 Hz), 6.24 (1H, d, *J* = 3.9 Hz), 6.13 (1H, s), 5.17 (2H, s), 4.44 (2H, t, *J* = 7.3 Hz), 4.20–4.17 (1H, m), 3.60 (1H, d, *J* = 5.6 Hz), 3.03 (2H, t, *J* = 7.3 Hz), 2.85–2.81 (2H, m), 2.57 (3H, s), 2.42–2.36 (2H, m), 2.29–2.18 (2H, m), 2.25 (3H, s), 1.92–1.81 (3H, m), 1.67–1.63 (1H, m), 1.60–1.54 (1H, m), 1.51–1.32 (4H, m), 0.80 (3H, s)
	^13^C NMR δ	160.6, 156.5, 156.1,144.4, 144.1, 138.0, 135.3, 133.2, 133.0, 128.2, 126.3, 123.9, 122.9, 120.6, 116.7, 114.8, 112.3, 89.9, 78.6, 62.0, 49.9, 47.8, 43.9, 43.8, 38.2, 36.6, 33.6, 29.7, 29.5, 27.2, 25.8, 25.7, 15.0, 12.3, 11.1
	HRMS	calcd for C_35_H_42_F_2_N_5_NaO_3_B [M + Na]^+^: *m/z* 651.3277. Found *m/z*: 651.3262

### HPLC Method Evaluation

Before evaluating our synthesized BODIPY-estrogen conjugates as fluorescent proxies in environmental bacteria, we first needed to establish reliable and robust analytical methods for concurrent quantitative analysis of both natural and fluorescent estrogens in culture media. First, the suitability of the chromatography system and method was evaluated to ensure that analyses of native and BODIPY-tagged estrogens were fairly compared. All estrogenic analytes were analyzed on the same run at two different wavelengths (230 nm for non-tagged species and 503 nm for BODIPY-tagged estrogens). Simultaneous analysis of native and fluorescent substrates allows for efficient sample processing and direct comparison of the overall chromatogram between samples. The analytes generated well-resolved (Rs > 1.5) Gaussian peaks (Figure S1). In fact, the chromatographic efficiency (*N*) was an order of magnitude greater for BODIPY-E1 and -E2 (*P* < 0.0025) than E1 and E2, respectively (Table S2). Thus, the developed method for concurrent analysis of natural and BODIPY-estrogens was deemed suitable for assessing analytical performance.

The chromatography method was then evaluated following the ICH Guidelines Q2 (R1) for analytical method validation and using the acceptance criteria set by the FDA Bioanalytical Method Validation Guidance.^41,42^ The ICH Guidelines describe “intermediate precision” as an expression of intra-laboratory variations, and this was investigated by comparing the response of a standard over a lengthy (>12 h) batch and between the first and final batches.^41^ The intermediate precision for all analytes not only satisfied the recommended acceptance criteria recommended by the FDA method validation guidelines (<15% RSD) but the non-derivatized estrogens had much higher variability compared to the BODIPY-estrogens (Table 3 and Figure S3).^42^

**Table 3:** Results of Method Evaluation^a^

	Precision			Accuracy		Calibration Statistics		
Analyte	Repeat.	Intra-Assay	Inter-Assay	% Recovery	% Error	LOD	LOQ	Linearity (*R*^2^)
E1	12.2	11.2	9.6	102.0	8.5	**0.09±0.03**	**0.27±0.09**	0.991 ± 0.005
						24 ± 8	72 ± 24	
E2	11.5	10.4	8.9	103.3	7.1	**0.10±0.04**	**0.30±0.13**	0.988 ± 0.009
						27 ± 12	82 ± 36	
BODIPY-E1	1.0	1.1	1.3	96.5	4.3	**0.02±0.01**	**0.07±0.03**	0.999 ± 0.001
						14 ± 7	42 ± 21	
BODIPY-E2	1.2	1.2	1.4	95.9	4.8	**0.02±0.01**	**0.07±0.03**	0.999 ± 0.001
						13 ± 6	40 ± 19	

a Precision values are percent relative standard deviation. The limit of detection (LOD) and limit of quantitation (LOQ) are reported in micromolar (**bold**, top values) and μg/L (bottom values). The linearity and limit values are the means of the three different batches (*n* = 3) and include standard deviation (±value).

The BODIPY-estrogens showed good quantitative accuracy compared to native estrogen HPLC analysis (Table 3, Figure S4). The percent recovery (eq S2) and percent error (eq S1) were generally within the recommended acceptance criteria: ±15% for the middle and high QC and ±20% for the lowest QC.^42^ In particular, the percent error for the BODIPY-estrogens was significantly less than those of the natural estrogens (Tables S3 and S4).

Unweighted linear regression of the calibration standards consistently showed a high degree of linearity across all platforms (from 0.988 ± 0.009 to 0.999 ± 0.001, *n* = 3). Additionally, the percent error for each calibration standard (Figure S2) was within ±15% (except for lowest standard which was within ±20%), both with and without BODIPY.

Last, the instrument limits of detection and quantitation were calculated from the calibration curve linear regression of each batch (eq S3).^41^ The calculated LOD and LOQ (Table 3) were lower for estrogens tagged with BODIPY (0.02 and 0.07 μM) than the non-tagged estrogens (0.09–0.10 and 0.27–0.30 μM).

Interestingly, the addition of BODIPY not only did not impede chromatographic analysis but it improved each analytical figure of merit compared to the untagged analytes. The conjugated fluorophore greatly increased the theoretical plate count for better efficiency, which in turn had beneficial effects on the signal-to-noise ratio and the relevant performance criteria. The precision, accuracy, linearity, and limits of detection of the BODIPY-estrogens are further improved due to the increased signal-to-noise ratio of the fluorophore, which has a greater molar absorptivity at 503 nm (nearly 10^5^ M^–1^ cm^–1^) compared to natural estrogens detected by UV (2000 M^–1^ cm^–1^).^43,44^ Indeed, BODIPY has previously been used to derivatize biomolecules such as thiols,^45^ fatty acids,^46^ and aliphatic aldehydes^47^ to enhance their chromatographic analysis. Thus, in addition to demonstrating that we have a reliable analytical method to measure the biodegradation of native and BODIPY-tagged estrogens, these results notably support the use of BODIPY derivatives for enhanced HPLC detection.

### LLE Method Development

The impact of the BODIPY fluorophore on the sample extraction method from bacterial culture media was then evaluated. BODIPY-estrogens present unique complications that can affect extraction, including low solubility and intermolecular interactions. Preliminary work with SPE using hydrophobic–lipophilic balance (HLB) sorbent in extracting BODIPY-estrogens by standard protocols for native estrogens showed inadequate recovery. Liquid–liquid extraction (LLE) was thus selected for sample preparation due to the simple process of chemical partitioning between two immiscible phases. In addition, because ionic strength can influence extraction, the LLE method was developed and optimized for minimal salt medium Modified Medium B (MMB).

**Figure 2:**
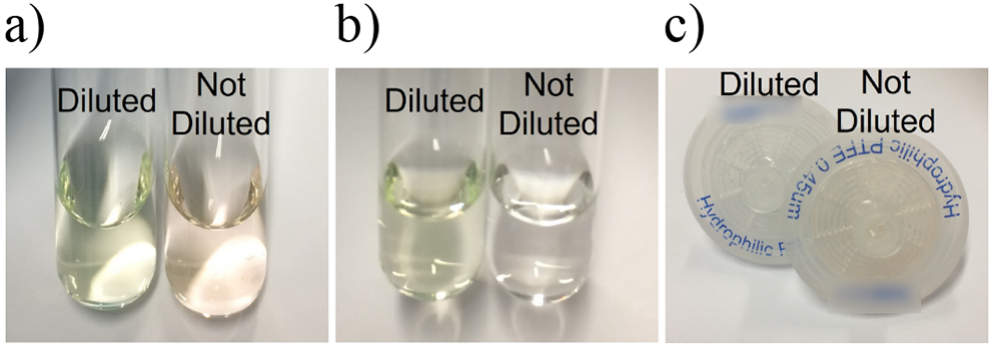
Photographs of estrogen and BODIPY-estrogen mixtures before (a) and after (b) filtration, showing the retention of green hue in the diluted mixture and loss of the orange hue in the undiluted mixture. (c) Syringe filters after filtration, showing an orange hue retained in the filter used on the undiluted mixture. The “diluted” mixture was combined with 50% (v/v) acetonitrile, and the “not diluted” mixture is in neat MMB media.

Filtration of biological samples is an important measure to preserve the integrity of the HPLC system and column, irrespective of the extraction method. However, syringe filtration appeared to remove the BODIPY compounds from solution. In an aqueous solution, the BODIPY-estrogens produce an orange hue (Figure 2a), in contrast to the vibrant green color observed in organic solvent such as acetonitrile or methanol. The orange color of BODIPY in aqueous solutions is understood to be the result of insoluble aggregates.^48^ The chromophoric BODIPY compounds were visually confirmed to have been filtered out of the media (Figure 2b) and retained in the filter material (Figure 2c). Therefore, to prevent the loss from filtration, the MMB medium was diluted 50% (v/v) with acetonitrile as it enabled solubilization of the BODIPY compounds (Figure 2a–c).

**Figure 3:**
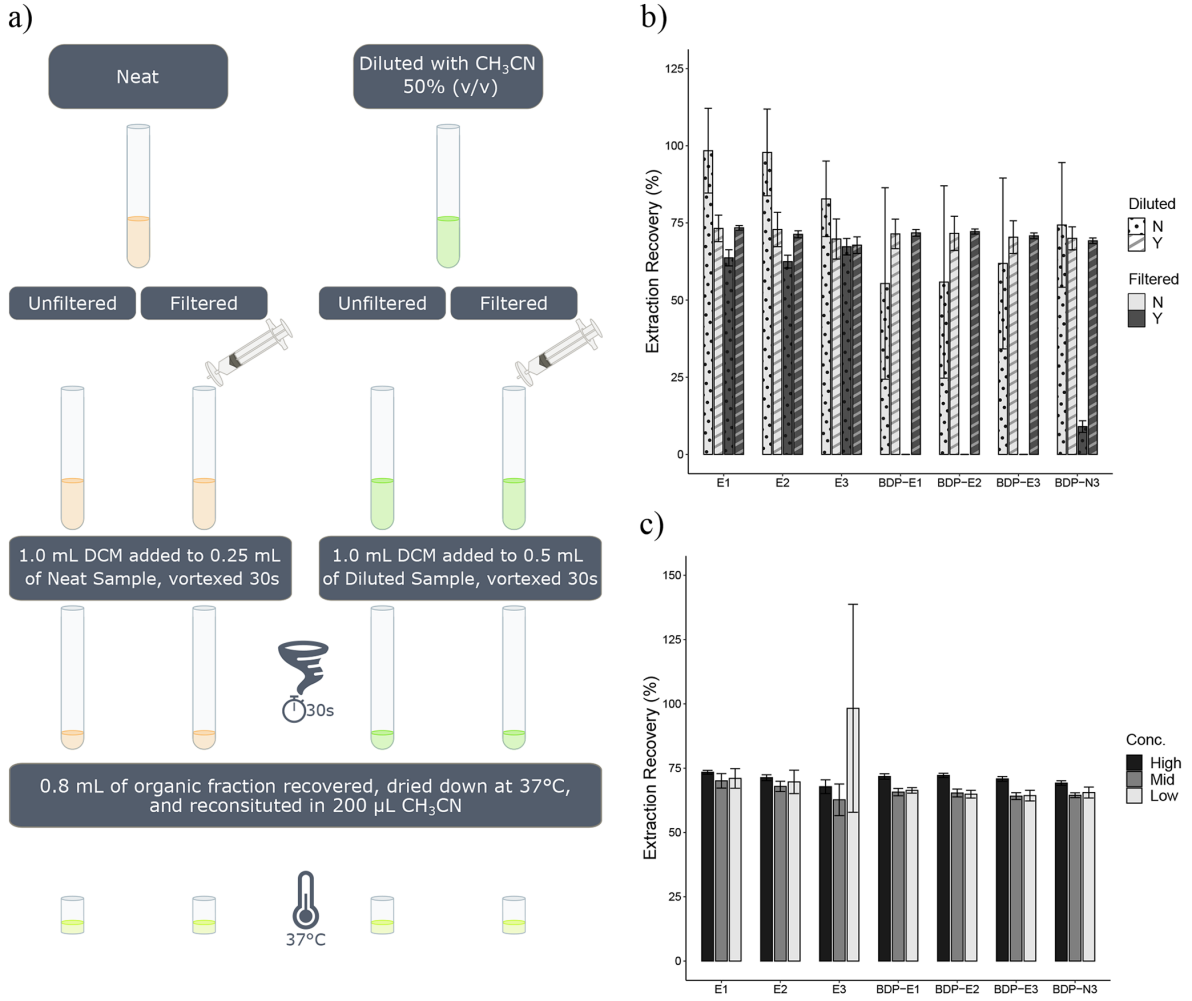
(a) Workflow of LLE method development. (b) Extraction recovery percentage of 1 mg/L estrogens and 0.5 mg/L BODIPY-azide by liquid–liquid extraction with (Y—stripes) and without dilution (N—dots) with acetonitrile and with (Y—dark gray) and without (N—light grey) filtration. (c) Extraction recovery percentage of estrogens and BODIPY azide with dilution and filtration. Low, mid, and high concentrations are 0.1, 0.5, and 1.0 mg/L for estrogens and 0.05, 0.25, and 0.5 mg/L for BODIPY-azide. Error bars represent the standard deviation of replicate samples extracted (*n* = 3).

The effects of dilution and filtration on the extraction recovery of natural and BODIPY-estrogens were quantitatively investigated (Figure 3a). BODIPY-azide was included in the recovery evaluation to assess the extraction performance on the fluorophore independently. The results of this work demonstrated that both processes have a significant impact on the extraction recovery of BODIPY compounds (Figure 3b). The samples filtered without dilution recovered only the natural estrogens. BODIPY-estrogens were not detected following extraction, and BODIPY-azide was only minimally recovered. Furthermore, the recovery of natural estrogens was poorer after filtering, indicating that filtration impedes extraction of natural estrogens as well. When the samples were diluted with acetonitrile prior to filtration, however, there was no difference in recovery between filtered and unfiltered samples for all compounds tested.

The extraction recovery for diluted and filtered samples across three concentrations (0.1, 0.5, and 1.0 mg/L each for natural and BODIPY-estrogens and 0.05, 0.25, and 0.5 mg/L for BODIPY-azide) was also determined to assess any concentration-dependent effects (Figure 3c and Table S5). The extraction recovery was highly reproducible across the concentrations of natural estrogens, except for the low concentration of E3. The high variability and difference from other estrogens may be explained by the lower response factor of E3, which has been reported previously in the literature.^49,50^ The extraction recovery performance was also highly reproducible across BODIPY compounds, including BODIPY-azide (average overall recovery 66.4–67.9%). The extraction recovery was slightly greater at the high concentration for BODIPY-estrogens and -azide, but within 7.3% difference of the middle and low concentrations. Last, the extraction recovery for natural and BODIPY-estrogens was comparable (average overall recovery 66.4–71.5%).

The BODIPY fluorophore had a significant impact on both filtration and phase partitioning. By evaluating BODIPY-azide along with the BODIPY-estrogens, it was clear that it was not the conjugation with steroids but the fluorophore itself that primarily affected the extraction recovery. We believe that this is directly related to the aggregative properties and low aqueous solubility of BODIPY. While there are extraction protocols for BODIPY-labeled lipids in the literature, these methods were not used for co-extracting the natural molecule.^51,52^ The extraction recoveries for the developed method were not as high as many published methods for natural estrogens.^53,54^ However, the method was reproducible across multiple estrogen species and a range of concentrations, both with and without the BODIPY tag. Thus, the measured concentrations, obtained by simultaneous extraction and analysis of natural and fluorescent estrogens, can be directly compared.

### Effects of HPβ-CDX on LLE

The low solubility of BODIPY-estrogens still presented a challenge for assessing biodegradation in aqueous biological samples. When prepared at 1 mg/L in MMB media, BODIPY-estrogens slowly (within 24 h) precipitate. Biodegradation experiments with high concentration of natural estrogens (0.5 g/L) were previously carried out by including methylated β-cyclodextrin.^18^ To ensure that the concentration in the solution remains consistent for the duration of experiments, HPβ-CDX was added to the minimal media to solubilize the BODIPY-estrogens.

**Figure 4:**
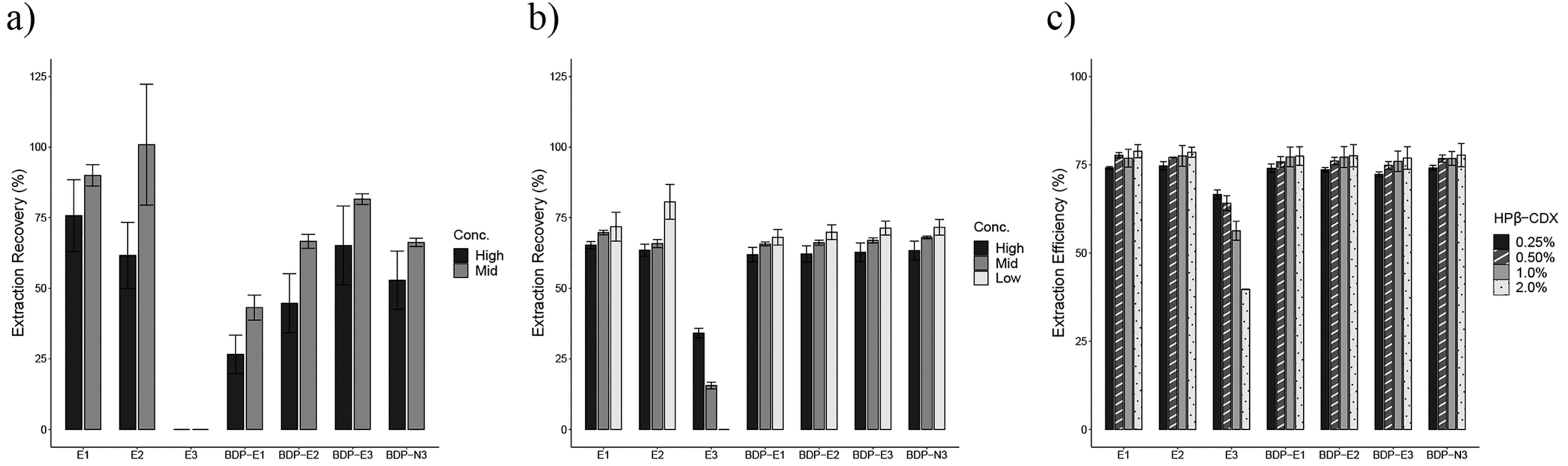
(a) Extraction recovery percentage of estrogens and BODIPY-azide in MMB media with 2% HPβ-CDX by LLE with filtration but without dilution. (b) Extraction recovery percentage of estrogens and BODIPY-azide in MMB media with 2% HPβ-CDX by LLE with dilution with acetonitrile and filtration. Low, mid, and high concentrations are 0.1, 0.5, and 1.0 mg/L for estrogens and 0.05, 0.25, and 0.5 mg/L for BODIPY-azide, respectively. Error bars represent the standard deviation of replicate samples extracted (*n* = 3). (c) Extraction efficiency percentage of 1 mg/L estrogens and 0.5 mg/L BODIPY-azide in MMB media with four different concentrations of HPβ-CDX by LLE without filtration or dilution. Error bars represent the standard deviation of replicate samples extracted (*n* = 2).

First, the recovery of natural and BODIPY-estrogens and BODIPY-azide was assessed in MMB media supplemented with 2% HPβ-CDX with filtration but without dilution as it was expected that cyclodextrin might replace the requirement for dilution (Figure 4a and Table S5). The results show that cyclodextrin has a notable effect on the extraction performance of all compounds. Most notably, the BODIPY-estrogens and BODIPY-azide were recovered (overall average recovery 34.9–73.4%), whereas these were not recoverable without HPβ-CDX (Figure 3b). Estriol, however, was not recovered in the presence of cyclodextrin following filtration. In addition, the extraction recovery was influenced by the analyte concentration and chemistry. Of the two concentrations investigated, the lower concentration consistently showed better extraction recovery. Additionally, the extraction recovery correlated negatively with hydrophobicity for the BODIPY compounds (BODIPY-E3 > BODIPY-azide > BODIPY-E2 > BODIPY-E1).

Next, the recovery was assessed including both cyclodextrin and 1:2 dilution with acetonitrile before filtration (Figure 4b and Table S5). The pre-dilution of the media supplemented with 2% HPβ-CDX with acetonitrile significantly diminished the combined effects of cyclodextrin and filtration as reported in Figure 4a. The extraction recovery was highly consistent across the natural estrogens and BODIPY compounds, again, with the exception of E3. Aside from estriol, there was a slight increase in extraction recovery at lower concentrations for all analytes tested, particularly for E2 (63.5% at 1.0 mg/L vs 80.6% at 0.1 mg/L). However, the overall average recovery for all estrogens (except E3) and BODIPY compounds fell between 65.2% and 70%.

Last, the influence of HPβ-CDX on the LLE process was investigated without dilution and filtration. Increasing concentrations of cyclodextrin minimally improved the extraction efficiency for all compounds investigated, except for E3 (Figure 4c). Increasing concentrations of cyclodextrin significantly reduced the extraction efficiency of E3. The markedly different extraction performance may be a consequence of the greater hydrophilicity of E3 compared to the other analytes investigated. However, the inverse effect was not observed as the strongly hydrophobic BODIPY-estrogens and BODIPY-azide showed a similar extraction efficiency to E1 and E2. Since there was no significant difference in extraction performance for most of the analytes tested at the concentrations of HPβ-CDX evaluated, the highest concentration of 2% (w/v) was selected to ensure the solubility of BODIPY-estrogens and potential metabolites.

Although cyclodextrin introduced some concentration-dependent effects and sacrificed E3, the developed LLE method reliably and simultaneously extracted the most natural and all BODIPY-tagged estrogens from minimal media without concern for solubility. Since the analyte concentration had a small effect on extraction recovery, it is not appropriate to apply a recovery factor to correct the analytical results as the recovery factor varies slightly by concentration.^55^ Furthermore, E3 cannot be used as a surrogate standard to correct the measured concentrations of E1 and E2 since the higher polarity of E3 makes it chemically incomparable during extraction. However, because the developed extraction method recovery is highly reproducible between natural and fluorescent estrogens, we can directly compare the biodegradation of both forms by extracting a control with the substrates spiked at the same concentration. Furthermore, if the fluorophore was deconjugated from the molecule, either enzymatically or abiotically, the now-untagged estrogen could be quantified in the very same extract. Because we have thoroughly evaluated the extraction and analysis methods, we can confidently measure the degradation of estrogens as well as their fluorescent analogues in bacterial cultures.

### Biodegradation of Estradiol versus BODIPY-Estradiol

After developing a robust analytical workflow to quantify native and BODIPY-labeled estrogens from culture media, we then assessed the use of the synthesized BODIPY-estrogens as surrogate substrates by comparing the degradation of 1 mg/L native E2 and BODIPY-E2 with known estrogen-degrading bacterium *C. tardaugens* DSM 16702. The full mineralization of E2 by *C. tardaugens* was fully characterized by Ibero et al. (2020). E2 is first oxidized to E1 by 17β-hydroxysteroid dehydrogenase, and subsequent transformations occur on the estrogen A-ring.^18^ Sodium pyruvate, a known growth substrate for DSM 16702,^18^ was included to determine if *C. tardaugens* requires an additional carbon and energy source to metabolize the BODIPY-tagged estrogen. The developed analytical workflow was used to measure and compare the concentrations of native E2 and BODIPY-E2 and expected metabolite E1, with and without the fluorescent tag.

**Figure 5:**
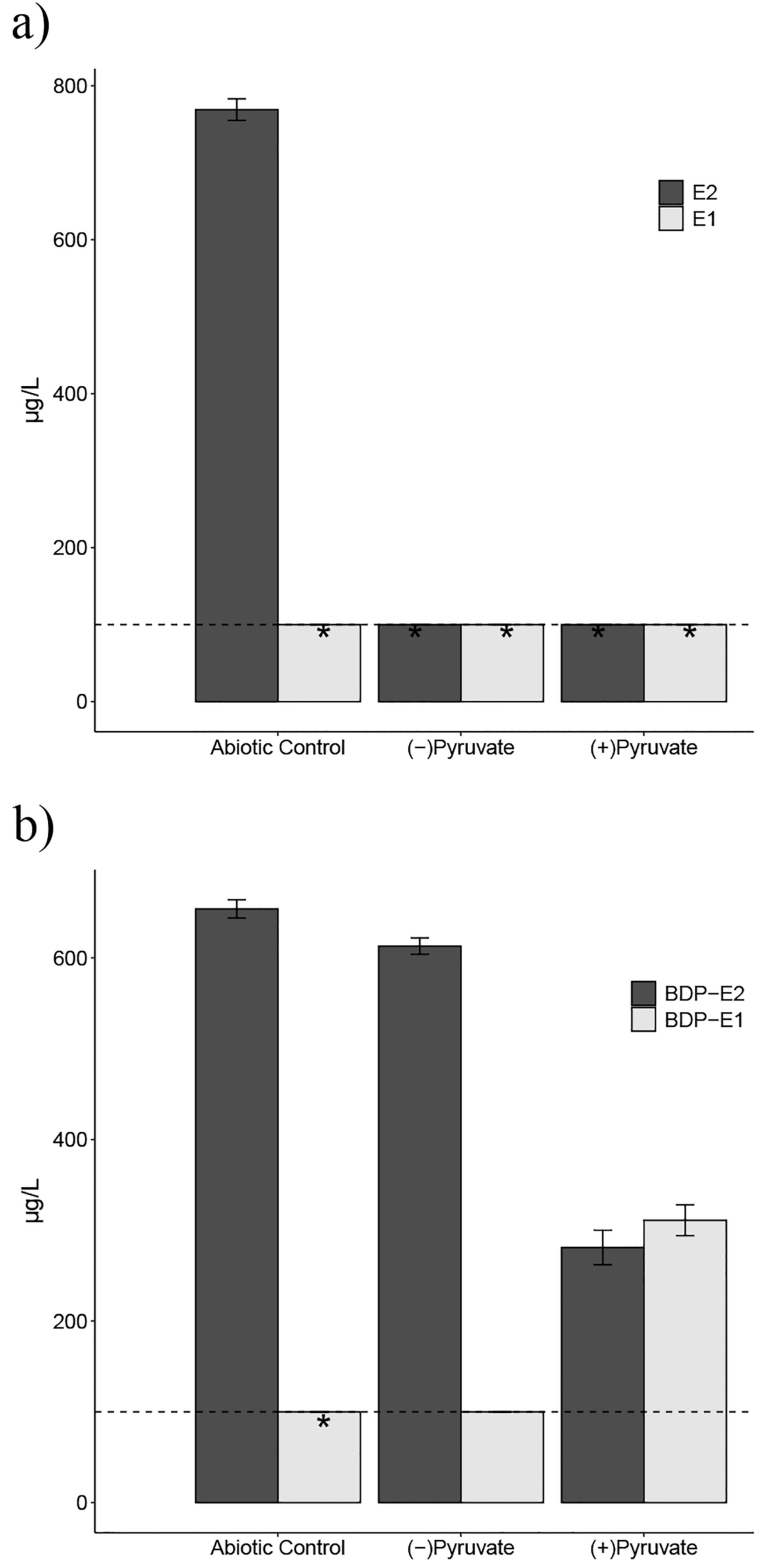
Concentrations of (a) E2 and E1 and (b) BODIPY-E2 and -E1 in DSM 16702 cultures after 1 week. Bars are the measured concentration in the abiotic control and duplicate biotic cultures with (+) or without (−) added sodium pyruvate. The dashed line represents the limit for accurate quantitation (lowest standard, 100 μg/L), and * indicates where the target analyte was not detected.

**Figure 6:**
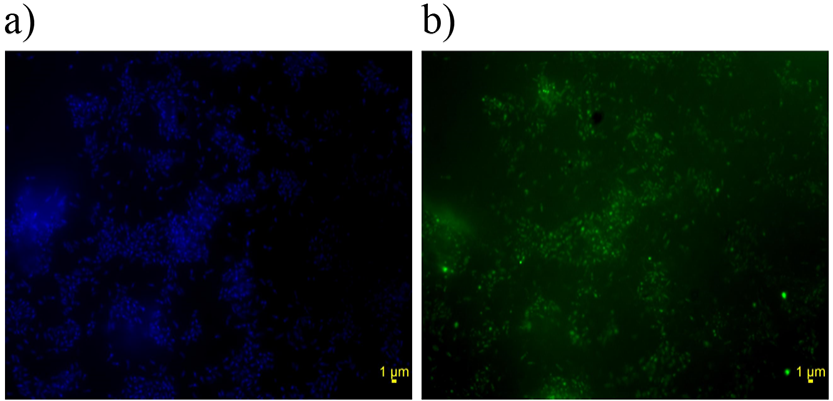
Fluorescent images of DSM 16702 cultured with 1 mg/L BODIPY-E2 for (a) DAPI (filter excitation 402 nm) and (b) BODIPY (filter excitation 470 nm).

The results showed complete degradation of natural E2 after 1 week, both with and without the pyruvate co-substrate (Figure 5a). Natural E1 was also consumed by the estrogen-mineralizing bacteria by the time of sampling and was undetectable. Without pyruvate, approximately 94% relative to the abiotic control of BODIPY-E2 remained in solution, with a detectable but not quantifiable amount of BODIPY-E1 present (Figure 5b). In the presence of pyruvate (Figure 5b), without correcting for recovery, 280 ± 20 μg/L BODIPY-E2 was measured after 1 week. Including BODIPY-E1 (310 ± 20 μg/L), the total fluorescent estrogens measured was slightly less than the concentration of BODIPY-E2 measured in the abiotic control (650 ± 10 μg/L). Although the BODIPY tag interfered with the downstream metabolism of estrogen and the metabolic rate, *C. tardaugens* successfully converted BODIPY-E2 to BODIPY-E1. Including pyruvate as a co-substrate greatly improved the biotransformation of BODIPY-E2 by providing a carbon and energy source to the bacteria. DSM 16702 cultured with BODIPY-E2, and pyruvate was further visualized by fluorescence microscopy. The BODIPY fluorescence (Figure 6b) was co-localized with the bacteria stained with DAPI (Figure 6a), demonstrating that the bacteria have taken on the fluorescent substrate.

We successfully confirmed the biotransformation of BODIPY-estradiol to BODIPY-estrone in a model estrogen-degrading bacterium originally isolated from a complex environmental sample, that is, activated sludge.^40^ We hypothesized that if *C. tardaugens* can metabolize BODIPY-E2, it would transform into BODIPY-E1 but no further because the fluorescent tag, which is conjugated at C3 of the estrogen A-ring, would sterically interfere with the remaining degradation steps. Additionally, we note that 2-hydroxypropyl-β-cyclodextrin is a suitable medium supplement to facilitate the solubility of BODIPY-estrogens, which does not appear to interfere with biodegradation. We confirmed these findings by chromatographic quantification of the native and fluorescent estrogen, supported by visual confirmation of the fluorescent substrate uptake using fluorescence microscopy. The synthesized BODIPY-E2 is a viable surrogate to investigate the uptake and biodegradation of estrogen by environmental microbes by fluorescence techniques.

## Conclusions

Fluorescent biological probes, particularly those based on the BODIPY framework, are often used to study biological processes in situ and in near real time. Fluorescent probes synthesized for environmental contaminants have the potential to enable fluorescence analysis of the uptake of these contaminants in environmental microbial communities and consequently faster discovery of new degrading bacteria compared to traditional enrichment methods. In turn, this can be used to synthetically design efficient microbiological remediation systems for environmental contaminants.

The important changes made to the molecule through the fluorescent labeling, such as molecular weight, availability of the sites of attacks, and solubility, however, can restrict the use of a probe. We demonstrated that a BODIPY-estradiol, synthesized using a simple click reaction with no modifications to the fluorophore structure for solubility or bioavailability, was taken up and transformed into the metabolite BODIPY-estrone by a well-characterized, model estrogen-degrading bacterium. The probe is, thus, ready to be deployed in environmental samples to observe, monitor, and isolate new uptaking bacteria. The click chemistry employed is also suitable for most environmental contaminants that contain labile hydrogens; the approach is therefore suitable beyond estrogenic molecules.

## Experimental Section

### Reagents

Estrogens estrone (≥99%), 17β-estradiol (≥98%), and estriol (≥97%) and HPLC-grade solvents were purchased from Sigma-Aldrich, UK. Ultrapure deionized water (18.2 MΩ cm, 0.22 μm filtered) was procured from a Milli-Q water system (Millipore). Individual stock solutions of each estrogen (tagged or non-tagged) were prepared in methanol at 1 mg/mL and stored in the dark at −20 °C.

The minimal salt medium, modified medium B (MMB; 10 mM (NH_4_)_2_SO_4_, 3 mM KH_2_PO_4_, 0.75 mM MgSO_4_·7H_2_O, 0.2 mM CaCl_2_·2H_2_O, 10 μM FeSO_4_·7H_2_O, 16 μM Na_2_EDTA·2H_2_O, 1 μM CuSO_4_·5H_2_O, 43 mM NaH_2_PO_4_·2H_2_O, 4 mM K_2_HPO_4_, and 0.04% Na_2_CO_3_), was prepared by dissolving salts in deionized water and adjusting to pH 8.0. The media was filter-sterilized and stored at room temperature. 2-Hydroxypropyl-β-cyclodextrin (average molecular weight 1460 Da, HPβ-CDX) was prepared as a filter-sterilized stock solution of 40% (w/v) in deionized water and diluted to the working concentration in MMB media.

### Synthesis of BODIPY-Estrogen Conjugates

The reactions were carried out in glassware dried in an oven (130 °C) and under an argon atmosphere. Tetrahydrofuran and dichloromethane were purified through a Pure Solv 400-5MD solvent purification system (Innovative Technology, Inc). All reagents were used as received, unless otherwise stated. Solvents were evaporated under reduced pressure at 40 °C. Column chromatography was performed under pressure using silica gel (Fluoro Chem Silica LC 60A) as the stationary phase. Reactions were monitored by thin-layer chromatography on aluminum sheets pre-coated with silica gel (Merck Silica Gel 60 F254). The plates were visualized by the quenching of UV fluorescence (λ_max_ 254 nm) and/or by staining with a KMnO_4_ solution or anisaldehyde dip.

Proton magnetic resonance spectra (^1^H NMR) and carbon magnetic resonance spectra (^13^C NMR) were recorded at 400 and 100 MHz or at 500 and 125 MHz using either a Bruker DPX Avance 400 instrument or a Bruker Avance III 500 instrument, respectively. IR spectra were obtained employing a Golden Gate with a type IIa diamond; thus, all the IR spectra were detected directly as thin layers without any sample preparation (Shimadzu FTIR-8400). Only significant absorptions are reported.

High-resolution mass spectra were recorded by the analytical group of the School of Chemistry at Glasgow University using a JEOL JMS-700 mass spectrometer by electrospray and chemical ionization operating at a resolution of 15,000 full widths at half-height. The complete details for the synthesis of azido-BODIPY tag and the derivatization of estrogens with BODIPY are described in Supporting Information I—Synthesis of BODIPY and BODIPY-Linked Estrogens.

### HPLC Analysis

Non-tagged and BODIPY-tagged estrogens were analyzed by the Prominence HPLC-PDA system (Shimadzu Corp.), which consisted of an inline degasser, a quaternary pump (LC-20AT), an autosampler, a column oven, and a photodiode array detector (SPD-M20A). The analytical column was a Purospher RP-18 (150 × 4.6 mm^2^, 5 μm pore) from Millipore and was maintained at 35 °C during analysis. The mobile phase consisted of ultrapure water (A) and acetonitrile (B) at a flow rate of 0.5 mL/min. The injected volume was 10 μL. Elution was carried out by an initial hold of 60% A:40% B for 3 min, followed by an increase to 100% B at +5% B per minute, then a purge at 100% B for 3 min before reconditioning at 60% A:40% B for 10 min (28 min total runtime). Non-derivatized estrogens were measured at 230 nm, which yielded a signal-to-noise ratio greater than 280 nm, the maximum absorbance wavelength determined by the detector (Table 1). BODIPY-tagged estrogens were measured at 503 nm, which was the maximum absorbance wavelength. Peak detection and integration were carried out using Shimadzu LabSolutions software.

### Standard Preparation

Six calibration standards and three quality control (QC) samples were prepared by diluting stock solutions in acetonitrile (see Supporting Information II—Standards Preparation, Table S1). A separate standard of the same concentrations as the top standard was used for determining system suitability and precision. Each standard and QC contained E3 and BODIPY-E3 as internal standards (IS) for detection at 230 nm and 503 nm, respectively.

### Liquid–Liquid Extraction

The extraction method was developed using MMB as the sample matrix. LLE was conducted in 15 mL glass test tubes by gently vortexing 0.25 mL of MMB spiked with estrogen mixture (1 mg/L each of E1, E2, E3, BODIPY-E1, BODIPY-E2, and BODIPY-E3 and 0.5 mg/L BODIPY-N3) with 1 mL of dichloromethane for 30 s. Exactly 0.8 mL of organic phase was recovered and evaporated to dryness at 37 °C before resuspending in 0.2 mL of acetonitrile. The analysis was conducted as described within the “HPLC Analysis” section.

When the spiked MMB was diluted 50:50 with acetonitrile prior to extraction, LLE was conducted by gently vortexing 0.5 mL of diluted sample with 1 mL of dichloromethane for 30 s. Filtered samples were prepared using a 0.45 μm hydrophilic PTFE syringe filter.

### System Suitability

The analytical methods were first evaluated for system suitability for each analyte. Six replicate measurements were used to determine theoretical plates and resolution. Resolution was calculated using the retention time and peak width and against the preceding analyte. The HPLC-PDA values for retention time (*t*_R_) and peak width at half-maximum (*W*_0.5_) were reported by Lab Solutions software.

Resolution (Rs) was calculated (eq 1) for a given analyte using the retention time and width at half-maximum and against the analyte preceding in order of elution, that is, where *t*_*R*2_ > *t*_*R*1_(56)
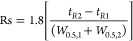
1

The theoretical plate number (*N*) was also calculated (eq 2) using the retention time and width at half-maximum for each analyte.^56^
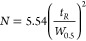
2

### Method Evaluation

The HPLC method was evaluated according to the International Conference on Harmonization (ICH) Guidelines Q2 (R1) for method validation in terms of specificity, precision, linearity, range, accuracy, and instrumental limits of detection and quantitation.^41^ The specific equations used for determining these figures of merit are given in Supporting Information IV—Method Evaluation Calculations and Results. Method evaluation was carried out by running a batch of six calibration standards and duplicate measurements of three QC samples on three separate days. On the first day, six replicate injections of a precision standard were also measured at the start and end of the batch. On the third day, six replicate injections of a precision standard were measured at the start of the batch for inter-assay reproducibility.

The extraction method was evaluated by relative recovery and efficiency. The relative recovery was determined from the ratio of the analyte peak area in the sample spiked before extraction versus the analyte peak area in the sample spiked after extraction and before evaporating. The extraction efficiency was determined from the ratio of the analyte peak area in the sample spiked before extraction versus the analyte peak area of a standard of the expected concentration.

### Biodegradation of Estradiol versus BODIPY-Estradiol

The biological transformation of E2 and BODIPY-E2 was evaluated in batch cultures of *C. tardaugens* DSM 16702 (Leibniz-Institut DSMZ, Germany). Prior to the biodegradation experiment, DSM 16702 was pre-cultured in LB medium for 4 days shaking at 150 rpm at 30 °C. The bacterium was harvested for the experiment by centrifuging at 2000*g* for 10 min and washing the bacterial pellet with 10 mL of MMB. The washed cell pellet was resuspended in fresh MMB to an optical density (600 nm) of 1.0 for the inoculum.

The experiment was conducted by inoculating 10 mL of MMB supplemented with 2% HPβ-CDX and 2.5 μg/mL thiamine HCl with 50 μL of *C. tardaugens* at OD_600_ 1.0. An equivalent volume of MMB media was used for the abiotic controls. The medium contained either 1 mg/L E2 or 1 mg/L BODIPY-E2. In addition, half of the cultures for each substrate were supplemented with 0.5 mg/mL sodium pyruvate to determine if a secondary carbon source was required for biodegradation of the BODIPY-tagged estrogen. Each test condition was cultured in duplicate for 1 week in the dark, shaking at 150 rpm at 30 °C.

At the conclusion of the experiment, a 1.5 mL sample of culture medium was mixed with an equal volume of acetonitrile (50:50 v/v) before filtering through a 0.45 μm PTFE syringe filter. Estrogens were extracted by gently vortexing 0.5 mL of filtrate with 1 mL of dichloromethane for 30 s. Exactly 0.8 mL of organic phase was recovered and evaporated to dryness at 37 °C before resuspending in 0.2 mL of acetonitrile. The extracts were stored at −20 °C in autosampler vials and analyzed within 48 h. The analysis was conducted as described within the “HPLC Analysis” section. The calibration standards were 100, 200, 400, 600, 800, and 1000 μg/L each of E1, E2, BODIPY-E1, and BODIPY-E2 and 1000 μg/L E3 and BODIPY-E3 as the internal standards for natural and fluorescent estrogens, respectively.

### Fluorescence Imaging of *C. tardaugens*

The fluorescence of DSM 16702 by uptake of BODIPY-E2 was captured by fluorescence microscopy. Bacteria were cultured for 48 h as per the “Biodegradation of Estradiol versus BODIPY-Estradiol” section with 1 mg/L BODIPY-E2 and 0.5 mg/mL sodium pyruvate. After incubation, 400 μL of culture was fixed with 500 μL of PBS and 100 μL 20% (w/v) paraformaldehyde. The fixed culture was filtered through a 0.2 μm Whatman Nuclepore polycarbonate filter. The filter was washed with 0.1% Triton X-100 in PBS and stained with 0.01 mg/mL DAPI Readymade Solution (Sigma-Aldrich UK) for 15 min. The filter was mounted on a slide with Fluoroshield Mounting Medium (Sigma-Aldrich UK) and immediately visualized with an Olympus IX-71 microscope at 100× magnification.

## Supporting Information Available

Details of the methodology and results pertaining to synthesis and method evaluation and NMR and HRMS spectra (PDF)

## Abbreviations

BODIPY: 4,4-difluoro-4-bora-3a,4a-diaza-*s*-indacene
E1: estrone
E2: 17β-estradiol
E3: estriol
HPLC: high-performance liquid chromatography
PDA: photodiode array detector
HPβ-CDX: 2-hydroxypropyl-β-cyclodextrin
