# Supplementary material for: BODIPY-Labeled Estrogens for Fluorescence Analysis of Environmental Microbial Degradation
Source: ACS Omega. 2022 Nov 3;7(45):41284–95. doi: 10.1021/acsomega.2c05002 (PMC9670910; doi:10.1021/acsomega.2c05002)
Supplement: Supplementary file 1 — ao2c05002_si_001.pdf [file ao2c05002_si_001.pdf]

Supporting Information

BODIPY-Labeled Estrogens for Fluorescence Analysis of Environmental Microbial Degradation

Celeste Felion[1], Ricardo Lopez-Gonzalez[2,3], Alan L. Sewell[2], Rodolfo Marquez[2,3], Caroline Gauchotte-Lindsay[1]

[1]James Watt School of Engineering, University of Glasgow, Glasgow G12 6EW, UK

[2]School of Chemistry, University of Glasgow, Glasgow, G12 8QQ, UK

[3]School of Physical and Chemical Sciences, University of Canterbury, Christchurch, 8140, New Zealand

*Corresponding author:

Celeste Felion

James Watt School of Engineering, University of Glasgow, Glasgow G12 6EW, UK

Email: c.felion.1@research.gla.ac.uk

Table of Contents

I. Synthesis of BODIPY and BODIPY-Linked Estrogens 2
II. Standards Preparation 11
III. System Suitability Results 12
IV. Method Evaluation Calculations and Results 13
a. Linearity 13
b. Precision 14
c. Accuracy 15
d. Instrument Limits of Detection and Quantitation 17
V. LLE Recovery Results 18
VI. BODIPY Estrogens NMR and HRMS Spectra 19
VII. References 31

I. Synthesis of BODIPY and BODIPY-Linked Estrogens

General. The reactions were carried out in glassware dried in an oven (130°C) and under an argon atmosphere. Tetrahydrofuran and dichloromethane were purified through a Pure Solv 400-5MD solvent purification system (Innovative Technology, Inc). All reagents were used as received, unless otherwise stated. Solvents were evaporated under reduced pressure at 40°C. Column chromatography was performed under pressure using silica gel (Fluoro Chem Silica LC 60A) as the stationary phase. Reactions were monitored by thin layer chromatography on aluminium sheets pre-coated with silica gel (Merck Silica Gel 60 F254). The plates were visualised by the quenching of UV fluorescence (λ_{max} 254 nm) and/or by staining with a $KMnO_4$ solution or anisaldehyde dip.

Proton magnetic resonance spectra (^{1}H NMR) and carbon magnetic resonance spectra (^{13}C NMR) were recorded at 400 MHz and 100 MHz or at 500 MHz and 125 MHz using either a Bruker DPX Avance 400 instrument or a Bruker Avance III 500 instrument, respectively. IR spectra were obtained employing a Golden Gate with a type IIa diamond, thus all the IR spectra were detected directly as thin layers without any sample preparation (Shimadzu FTIR-8400). Only significant absorptions are reported.

High resolution mass spectra were recorded by the analytical group of the School of Chemistry at Glasgow University using a JEOL JMS-700 mass spectrometer by electrospray and chemical ionisation operating at a resolution of 15,000 full widths at half height.

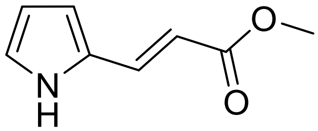

Methyl (*E*)-3-(1*H*-pyrrol-2-yl)acrylate, X1.

A solution of 1*H*-Pyrrole-2-carboxaldehyde (2.0 g, 21.0 mmol) in benzene (160 mL) was treated with methyl (triphenylphosphoranylidene)acetate (10.9 g, 32.6 mmol) and then refluxed for 18 h. The reaction was then cooled down to rt and the solvent was removed *in vacuo* to give a crude yellow oil. Purification of the crude residue by flash column chromatography (0-20% EtOAc/PE) gave the desired ester **X1** as a white solid (2.3 g, 73%). The NMR data obtained is in agreement with the literature data.[1]

^{1}H NMR ($CDCl_3$, 400 MHz) δ: 8.93 (1H, br s), 7.58 (1H, d, J = 16.0 Hz), 6.97–6.94 (1H, m), 6.58–6.57 (1H, m), 6.32–6.29 (1H, m), 6.06 (1H, d, J = 16.0 Hz), 3.80 (3H, s). ^{13}C NMR ($CDCl_3$, 100 MHz) δ: 168.0, 134.3, 128.3, 122.4, 114.5, 111.0, 110.8, 51.5.

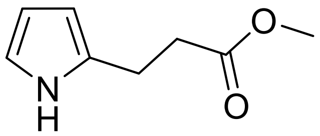

Methyl 3-(1*H*-pyrrol-2-yl)propanoate, X2.

Methyl (*E*)-3-(1*H*-pyrrol-2-yl)acrylate **X1** (2.3 g, 15.2 mmol) was dissolved in MeOH (100 mL), and placed under at atmosphere of argon. Pd/C 10 wt. % (240 mg, 10 mol%) was added and then the argon atmosphere was replaced with a hydrogen atmosphere before stirring the reaction at rt for 16 h. The crude mixture was the filtered over a bed of celite which was then washed with MeOH (2 x 25 mL). The combined washes were concentrated under reduced pressure to afford the desired ester **X2** (2.2 g, 93%) as a pale yellow oil, which required no further purification. The NMR data obtained is in agreement with the literature data.[1]

^{1}H NMR ($CDCl_3$, 400 MHz) δ: 8.54 (1H, br s), 6.71–6.69 (1H, m), 6.14–6.11 (1H,m), 5.96–5.93 (1H, m), 3.72 (3H, s), 2.94 (2H, t, J = 8.0 Hz), 2.67 (2H, t, J = 8.0 Hz). ^{13}C NMR ($CDCl_3$, 100 MHz) δ: 174.5, 130.9, 116.8, 108.0, 105.5, 51.8, 34.3, 22.5.

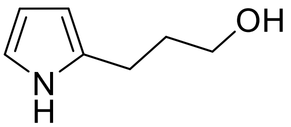

3-(1*H*-Pyrrol-2-yl)propan-1-ol, X3.

A solution of methyl 3-(1*H*-pyrrol-2-yl)propanoate **X2** (2.1 g, 13.7 mmol) in Et_2O (110 mL) was cooled down to 0 °C before being treated by the slow addition of $LiAlH_4$ (1.0 g, 26.4 mmol). The resulting suspension was stirred for 16 h whilst allowing it to warm up to rt. The crude reaction mixture was then quenched with 1M NaOH solution dropwise until pH neutral. The Et_2O phase was then decanted off, and the lithium/aluminium salts were washed with Et_2O (2 x 100 mL). The combined Et_2O phases were dried (Na_2SO_4), and the solvent removed *in vacuo* to give the desired alcohol **X3** (1.7 g, 100%) as a colourless oil, which required no further purification. The NMR data obtained is in accordance with the literature data.[1]

^{1}H NMR ($CDCl_3$, 400 MHz) δ: 8.16 (1H, br s), 6.72-6.68 (1H, m), 6.18-6.13 (1H, m), 5.98-5.94 (1H, m), 3.74 (2H, t, J = 6.0 Hz), 2.76 (2H, t, J = 8.0 Hz), 2.00-1.80 (2H, m), 1.50 (1H, br s). ^{13}C NMR ($CDCl_3$, 100 MHz) δ: 130.9, 116.4, 108.4, 105.3, 63.7, 28.9, 24.0.

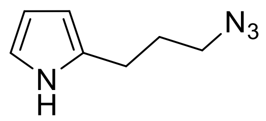

2-(3-Azidopropyl)-1*H*-pyrrole, X4.

A solution of 3-(1*H*-pyrrol-2-yl)propan-1-ol **X3** (1.0 g, 8.1 mmol) in CH_2Cl_2 (60 mL) was cooled down to 0 °C before the sequential addition of Et_3N (2.3 mL, 16.3 mmol) and methanesulfonyl chloride (755 µL, 9.8 mmol). The reaction mixture was then stirred for 1 h at 0 °C, before allowing it to warm up to rt. The reaction was then treated with aq. 1M HCl solution (40 mL), followed by aq. Sat. $NaHCO_3$ (60 mL) and brine (60 mL). The organic phase was dried (Na_2SO_4), and solvent removed *in vacuo* to afford the crude mesylate intermediate (1.5 g, 94%). The crude mesylate was then dissolved in DMF (60 mL), and the solution treated with sodium azide (1.5 g, 22.7 mmol). The reaction mixture was then heated to 70 °C until completion by TLC analysis (16 h). The reaction mixture was then cooled down to rt, and diluted with EtOAc (60 mL) followed by H_2O (60 mL). The layers were separated, and the aqueous phase was washed with EtOAc (2 x 60 mL). The combined organic layers were washed with brine (5 x 100 mL), dried (Na_2SO_4), and the solvent removed under reduced pressure to afford azide **X4** (947 mg, 88%) as a yellow oil, which required no further purification. The NMR data obtained is in accordance with the literature data.[1]

^1H NMR ($CDCl_3$, 400 MHz) δ: 7.98 (1H, br s), 6.70-6.68 (1H, m), 6.15-6.13 (1H, m), 5.96-5.94 (1H, m), 3.34 (2H, t, J = 6.6 Hz), 2.73 (2H, t, J = 7.4 Hz), 1.92-1.87 (2H, m). ^{13}C NMR ($CDCl_3$, 100 MHz) δ: 130.7, 116.5, 108.5, 105.5, 50.7, 28.9, 24.7.

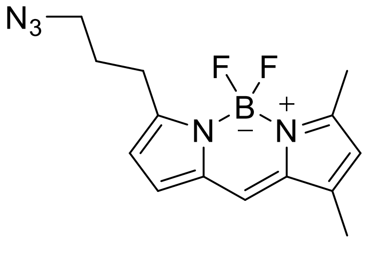

3-Azido[4,4-difluoro-5,7-dimethyl-4-bora-3a,4a-diaza-*s*-indacene-3-yl]propane, X5.

A solution of 2-(3-azidopropyl)-1*H*-pyrrole **X4** (270 mg, 1.8 mmol) and 3,5-dimethyl-1*H*-pyrrole-2-carboxaldehyde (203 mg, 1.6 mmol) in CH_2Cl_2 (12 mL) was cooled down to 0°C before being treated by the dropwise addition of $POCl_3$ (100 µL, 1.0 mmol). The mixture was allowed to warm up to rt and was stirred for 6.5 h before being cooled back down again to 0°C. After cooling, the reaction mixture was treated by the sequential addition of $BF_3.Et_2O$ (500 µL, 4.0 mmol) and DIPEA (700 µL, 4.0 mmol). The finally allowed to warm up to rt, and was stirred for a further 16 h. The reaction was quenched with H_2O

(15 mL), and diluted CH_2Cl_2 (5 mL) before being filtered through a bed of celite which was then washed thoroughly with CH_2Cl_2 (2 x 10 mL). The combined organic phases were dried (Na_2SO_4), and the solvent removed *in vacuo* to afford a crude dark red solid. Purification of the crude residue by flash column chromatography (20% EtOAc/petroleum ether) afforded the desired azido-BODIPY **X5** (290 mg, 59%) as a red oil which solidified upon cooling. The NMR data obtained is in accordance with that in the literature.[1]
^{1}H NMR ($CDCl_3$, 400 MHz) δ: 7.11 (1H, s), 6.94 (1H, d, J = 4.0 Hz), 6.31 (1H, d, J = 4.0 Hz), 6.14 (1H, s), 3.41 (2H, t, J = 8.0 Hz), 3.07 (2H, t, J = 8.0 Hz), 2.59 (3H, s), 2.28 (3H, s), 2.06-2.01 (2H, m).^{13}C NMR ($CDCl_3$, 100 MHz) δ: 160.5, 156.5, 146.7, 143.7, 134.1, 128.3, 123.8, 120.2, 116.6, 50.9, 28.2, 25.8, 14.9, 11.4.

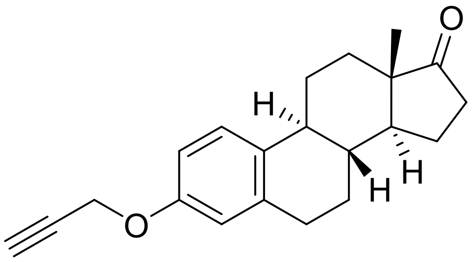

3-*O*-Propargylestrone, X6.
A solution of estrone (300 mg, 1.1 mmol) in DMF (7 mL) was treated with K_2CO_3 (768 mg, 5.5 mmol) in one portion. The resulting mixture was then treated with propargyl bromide (618 µL, 5.6 mmol, 80% in toluene) and the reaction was stirred at 70°C for 16 h. The reaction was cooled down to rt, and was then diluted with EtOAc (60 mL) before being washed with aq. satd $NaHCO_3$ (50 mL), H_2O (50 mL) and brine (5 x 30 mL). The organic phase was dried (Na_2SO_4), and the solvent was removed under reduced pressure to afford a crude yellow oil. Purification of the crude residue by flash column chromatography (10-20% EtOAc/ petroleum ether) afforded 3-*O*-propargylestrone **X6** (295 mg, 86%) as a yellow solid. The NMR data obtained is in accordance with the literature data.[2]
^{1}H NMR ($CDCl_3$, 400 MHz) δ: 7.23 (1H, d, J = 8.6 Hz), 6.80 (1H, dd, J = 8.6, 2.8 Hz), 6.73 (1H, d, J = 2.8 Hz), 4.67 (2H, d, J = 2.4 Hz), 2.94–2.90 (2H, m), 2.54–2.48 (1H, m), 2.52 (1H, t, J = 2.4 Hz), 2.23–1.43 (12H, m), 0.92 (3H, s). ^{13}C NMR ($CDCl_3$, 100 MHz) δ: 220.6, 155.6, 137.9, 133.0, 126.4, 115.0, 112.4, 78.8, 75.3, 55.8, 50.5, 48.0, 44.0, 38.3, 35.9, 31.6, 29.7, 26.5, 25.9, 21.6, 13.9.

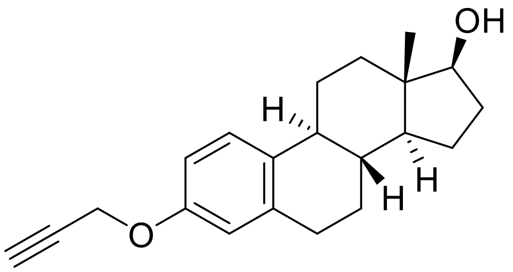

3-*O*-Propargyl-estradiol, X7.

3-*O*-Propargylestrone **X6** (100 mg, 0.3 mmol) was dissolved in a mixture of CH_2Cl_2 (2.5 mL), MeOH (2.5 mL) and H_2O (500 µL), and the resulting solution was treated wiith $NaBH_4$ (37 mg, 1 mmol) in one portion. The resulting mixture was then stirred at rt for 2h, and was then concentrated *in vacuo*. The crude residue was dissolved in CH_2Cl_2 (10 mL), and washed sequentially with 1M HCl (10 mL), H_2O (10 mL) and brine (10 mL). The organic layer was then dried (Na_2SO_4), and concentrated *in vacuo* to afford the estradiol product **X7** (86 mg, 86%) as a colourless oil, which required no further purification.

^{1}H NMR ($CDCl_3$, 400 MHz) δ: 7.23 (1H, d, J = 8.6 Hz), 6.80 (1H, dd, J = 8.6, 2.8 Hz), 6.71 (1H, d, J = 2.8 Hz), 4.67 (2H, d, J = 2.4 Hz), 3.74 (1H, t, J = 8.6 Hz), 2.90–2.84 (2H, m), 2.51 (1H, t, J = 2.4 Hz), 2.35–2.29 (1H, m), 2.23–2.09 (2H, m), 1.98–1.86 (2H, m), 1.75–1.67 (1H, m), 1.53–1.17 (7H, m), 0.79 (3H, s). ^{13}C NMR ($CDCl_3$, 100 MHz) δ: 155.5, 138.1, 133.7, 126.4, 115.0, 112.3, 81.9, 78.9, 75.3, 55.8, 50.1, 44.0, 43.3, 38.8, 36.7, 30.6, 29.8, 27.2, 26.3, 23.1, 11.1. IR υ_{max} (film)/cm^{-1} 3582, 3289, 2949, 2912, 2866, 2121, 1496. HRMS (ESI) calcd for $C_{21}H_{26}O_2Na$ $[M+Na]^+$: *m/z* 333.1825, found *m/z* 333.1810.

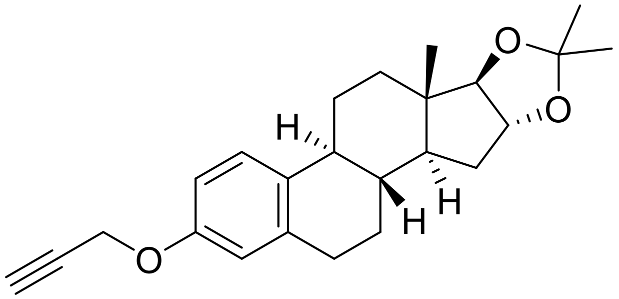

3-*O*-Propargyl- (16-*O*,17-*O*-dimethylacetyl)estriol, X8.

A suspension of estriol (100 mg, 0.3 mmol) in a mixture of THF (2 mL) and acetone (2 mL) was treated with *p*-TsOH (3 mg, 0.02 mmol), and the reaction was cooled to 0 °C before being treated with 2-methoxypropene (67 µL, 0.7 mmol). After 2h, a further portion of 2-methoxypropene (67 µL, 0.7 mmol) was added, causing the suspension to become a yellow solution. After stirring for a further 2 h at rt, the reaction mixture was neutralised with enough drops of Et_3N to turn the solution a pale yellow colour. The solution was dried (Na_2SO_4), and concentrated *in vacuo* to afford a crude yellow oil. Purification of the

crude residue by flash column chromatography (5-10% EtOAc/petroleum ether) afforded the ketal-protected product (90 mg, 79%) as a semi-crude compound. The semi-crude material (90 mg, 0.3 mmol) was dissolved in DMF (4 mL) and treated with K_2CO_3 (189 mg, 1.4 mmol). Propargyl bromide (153 µL, 1.4 mmol, 80% in toluene) was then added and the resulting reaction mixture was heated at 70 °C for 16h. The reaction was then cooled down to rt, and diluted with EtOAc (25 mL) before being washed with aq. Satd $NaHCO_3$ (20 mL), H_2O (20 mL) and brine (5 x 20 mL). The organic phase was dried (Na_2SO_4), and concentrated under reduced pressure to yield a crude yellow oil. Purification of the crude residue by flash column chromatography (10-20% EtOAc/petroleum ether) afforded 3-*O*-propargyl-(16-O, 17-O-dimethylacetyl)estriol **X8** (60 mg, 60% over two steps) as a colourless oil.

^{1}H NMR ($CDCl_3$, 500 MHz) δ: 7.22 (1H, d, *J* = 8.6 Hz), 6.78 (1H, dd, *J* = 8.6, 2.8 Hz), 6.70 (1H, d, *J* = 2.8 Hz), 4.66 (2H, d, *J* = 2.4 Hz), 4.22-4.20 (1H, m), 3.78 (1H, d, *J* = 4.7 Hz), 2.87–2.84 (2H, m), 2.51 (1H, t, *J* = 2.4 Hz), 2.29–2.22 (2H, m), 1.87–1.73 (3H, m), 1.58–1.18 (6H, m), 1.41 (3H, s), 1.35 (3H, s), 0.78 (3H, s). ^{13}C NMR ($CDCl_3$, 125 MHz) δ: 155.4,138.1, 133.7, 126.3, 114.9, 112.3, 100.0, 87.5, 78.9, 77.0, 75.3, 55.8, 47.7, 43.8, 43.0, 38.3, 37.2, 30.6, 29.8, 27.1, 26.9, 26.3, 26.0, 12.9. HRMS (ESI) calcd for $C_{24}H_{31}O_3$ $[M+H]^+$: *m/z* 367.2268, found *m/z* 367.2251.

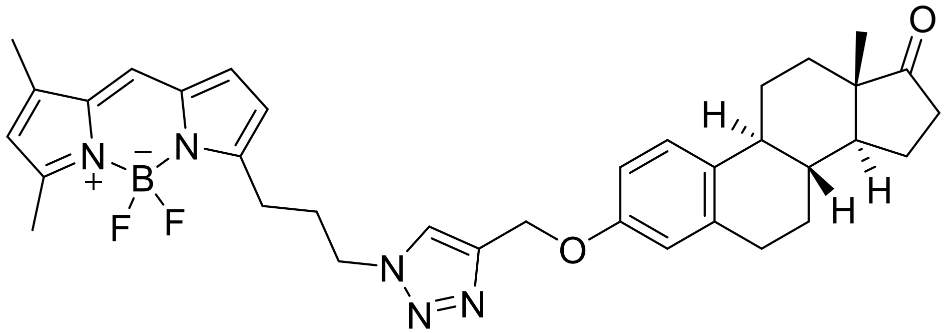

3-*O*-((3-[4,4-Difluoro-5,7-dimethyl-4-bora-3^a,4^a-diaza-*s*-indacene-3-yl]7ropil)-1*H*-1,2,3-triazol-4-yl)methoxy)estrone, X9.

A rt mixture of 3-*O*-propargylestrone **X6** (85 mg, 0.3 mmol) and 3-azido[4,4-difluoro-5,7-dimethyl-4-bora-3a,4a-diaza-*s*-indacene-3-yl]propane **X5** (84 mg, 0.3 mmol) in THF (5 mL) was treated with one drop of DIPEA, followed by catalytic amount of CuI (5 mg). The resulting mixture was then heated up to 70°C, and stirred for 18h. The reaction was cooled down to rt, and was then concentrated *in vacuo* to give a crude red oil. Purification of the crude residue by flash column chromatography (0-2% MeOH/CH_2Cl_2) afforded the desired compound **X9** (132 mg, 80%) as a red foam.

^{1}H NMR ($CDCl_3$, 500 MHz) δ: 7.63 (1H, s), 7.20 (1H, d, *J* = 8.5 Hz), 7.09 (1H, s), 6.88 (1H, d, *J* = 4.0 Hz), 6.79 (1H, dd, *J* = 8.5, 2.7 Hz), 6.73 (1H, d, *J* = 2.7 Hz), 6.24 (1H, d, *J* = 4.0 Hz), 6.13 (1H, s), 5.18 (2H, s), 4.43 (2H, t, *J* = 7.3 Hz), 3.04 (2H, t, *J* = 7.3 Hz), 2.91–2.87 (2H, m), 2.57 (3H, s), 2.53–2.47 (1H, m), 2.42–

2.36 (3H, m), 2.25 (3H, s), 2.25–1.94 (5H, m), 1.71–1.41 (6H, m), 0.91 (3H, s). ^{13}C NMR ($CDCl_3$, 125 MHz) δ: 220..6, 160.3, 156.6,156.3, 144.4, 144.1, 137.9, 135.3, 133.2, 132.6, 128.2, 126.4, 123.9, 122.8, 120.6, 116.7, 114.8, 112.4, 62.1, 50.4, 49.8, 48.0, 44.0, 38.3, 35.9, 31.6, 29.6, 29.5, 26.5, 25.9, 25.7, 21.6, 15.0, 13.9, 11.3. IR υ_{max} (film)/cm^{-1} 2933, 2861, 1731, 1600. HRMS (ESI) calcd for $C_{35}H_{40}F_2N_5NaO_2B$ $[M+Na]^+$: *m/z* 633.3172, found *m/z* 633.3162.

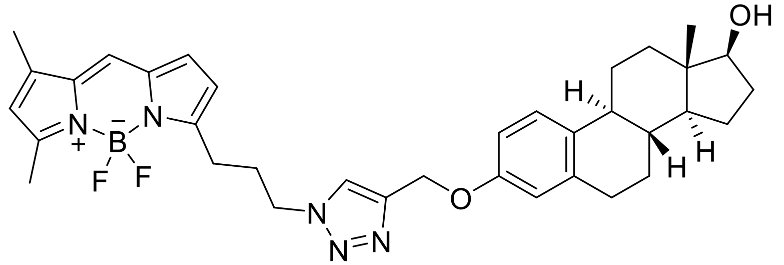

3-*O*-((3-[4,4-Difluoro-5,7-dimethyl-4-bora-3a,4a-diaza-*s*-indacene-3-yl]propyl)-1*H*-1,2,3- triazol-4 -yl) methoxy)!-estradiol, X10

A rt mixture of 3-*O*-propargyl-estradiol **X7** (69 mg, 0.2 mmol) and 3-azido[4,4-difluoro-5,7-dimethyl-4-bora-3a,4a-diaza-*s*-indacene-3-yl]propane **X5** (67 mg, 0.2 mmol) in THF (4 mL) was treated with one drop of DIPEA, followed by a catalytic amount of CuI (5 mg). The resulting mixture was then heated up to 70°C, and stirred for 18h. The reaction was then cooled down to rt, and was then concentrated *in vacuo* to give a crude red oil. Purification of the crude residue by flash column chromatography (0-2% MeOH/CH_2Cl_2) afforded the desired compound **X10** (117 mg, 86%) as a red foam.

^{1}H NMR ($CDCl_3$, 500 MHz) *δ*: 7.63 (1H, s), 7.20 (1H, d, *J* = 8.6 Hz), 7.09 (1H, s), 6.88 (1H, d, *J* = 3.9 Hz), 6.78 (1H, dd, *J* = 8.6, 2.7 Hz), 6.71 (1H, d, *J* = 2.7 Hz), 6.24 (1H, d, *J* = 3.9 Hz),6.13 (1H, s), 5.17 (2H, s), 4.43 (2H, t, *J* = 7.3 Hz), 3.73 (1H, t, *J* = 8.6 Hz), 3.04 (2H, t, *J* = 7.3 Hz), 2.87–2.83 (2H, m), 2.57 (3H, s), 2.42-2.36 (1H, m), 2.32–2.08 (3H, m), 2.25 (3H, s), 1.97–1.93 (1H, m),1.90–1.85 (1H, m), 1.73–1.67 (1H, m), 1.52–1.16, (9H, m), 0.78 (3H, s); ^{13}C NMR ($CDCl_3$,125 MHz) *δ*: 160.6, 156.6, 156.2, 144.4, 144.1, 138.1,135.4, 133.2, 133.5, 128.2, 126.4, 123.8, 122.8, 120.5, 116.7, 114.8, 112.3, 81.9, 62.1, 50.1, 49.8, 44.0, 43.3, 38.8, 36.7, 30.6, 29.8, 29.5, 27.2, 26.3, 25.7, 23.1, 15.0, 11.3, 11.1. IR υ_{max} (film)/cm^{-1} 2923, 2865, 1602. HRMS (ESI) calcd for $C_{35}H_{42}F_2N_5NaO_3B$ $[M+Na]^+$: *m/z* 633.3172, found *m/z* 633.3162.

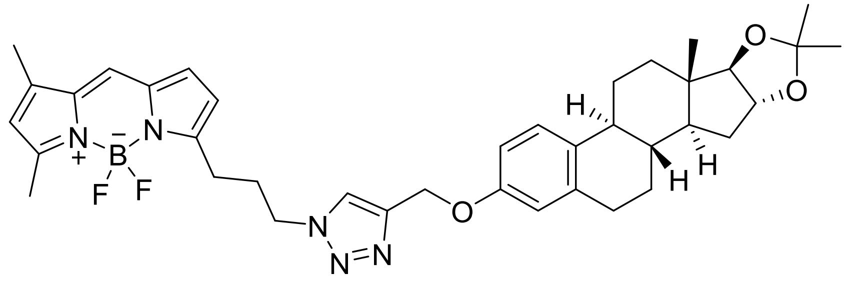

3-*O*-((3-[4,4-Difluoro-5,7-dimethyl-4-bora-3a,4a-diaza-*s*-indacene-3-yl]propyl)-1*H*-1,2,3-triazol-4-yl) methoxy)-(16-O,17-O-dimethylacetyl)estriol, X11

A rt mixture of 3-*O*-propargyl-(16-*O*,17-*O*-dimethylacetyl)estriol **X8** (58 mg, 0.2 mmol) and 3-azido[4,4-difluoro-5,7-dimethyl-4-bora-3a,4a-diaza-*s*-indacene-3-yl]propane **X5** (48 mg, 0.2 mmol) in THF (3 mL) was treated with one drop of DIPEA, followed by a catalytic amount of CuI (5 mg). The resulting mixture was then heated up to 70°C, and stirred for 18 h. The reaction was then cooled down to rt and concentrated *in vacuo* to give a crude red oil. Purification of the crude residue by flash column chromatography (0-2% MeOH/CH_2Cl_2) afforded the desired compound **X11** (54 mg, 51%) as a red oil.

^{1}H NMR ($CDCl_3$, 500 MHz) δ: 7.63 (1H, s), 7.19 (1H, d, J = 8.6 Hz), 7.09 (1H, s), 6.88 (1H, d, J = 3.9 Hz), 6.77 (1H, dd, J = 8.6, 2.5 Hz), 6.70 (1H, d, J = 2.5 Hz), 6.24 (1H, d, J = 3.9 Hz), 6.13 (1H, s), 5.17 (2H, s), 4.43 (2H, t, J = 7.3 Hz), 4.22–4.20 (1H, m), 3.77 (1H, d, J = 4.6 Hz), 3.03 (2H, t, J = 7.3 Hz), 2.85–2.81 (2H, m), 2.57 (3H, s), 2.42-2.36 (2H, m), 2.27–2.20 (2H, m), 2.25 (3H, s), 1.86–1.75 (3H, m), 1.58–1.35 (12H, m), 0.77 (3H, s). ^{13}C NMR ($CDCl_3$, 125 MHz) δ: 160.5, 156.6, 156.1, 144.4, 144.1, 138.1, 135.3, 133.2, 133.1, 128.2, 126.3, 123.9, 122.8, 120.6, 116.7, 114.8, 112.2, 100.0, 87.5, 77.0, 62.1, 49.9, 47.7, 43.8, 43.0, 38.3, 37.2, 35.4, 29.8, 29.5, 27.2, 26.9, 26.3, 26.0, 25.7, 15.0, 12.9, 11.1. HRMS (ESI) calcd for $C_{38}H_{46}F_2N_5O_3B$ $[M]^+$: *m/z* 668.3698, found *m/z* 668.3677.

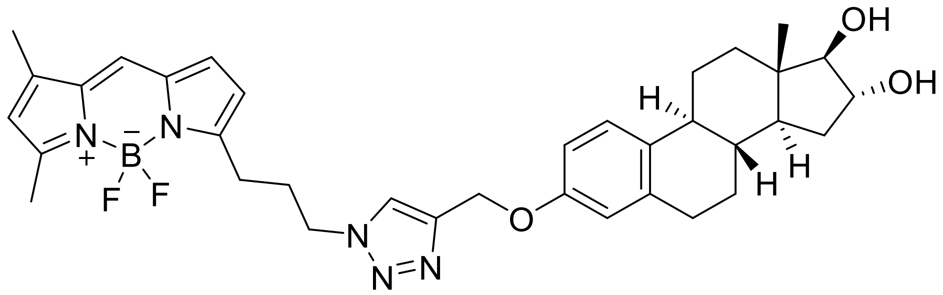

3-*O*-((3-[4,4-Difluoro-5,7-dimethyl-4-bora-3a,4a-diaza-*s*-indacene-3-yl]propyl)-1*H*-1,2,3- triazol-4-yl)methoxy)estriol, X12.

A solution of 3-*O*-((3-[4,4-Difluoro-5,7-dimethyl-4-bora-3a,4a-diaza-*s*-indacene-3-yl]propyl)-1*H*-1,2,3-triazol-4-yl)methoxy)-(16-*O*,17-*O*-dimethylacetyl)estriol **X11** (52 mg, 0.1 mmol) in MeCN (2 mL) was treated with H_2O (0.150 mL), followed by $BiCl_3$ (3 mg, 10 µmol) to give a cloudy red suspension. After 3h, the reaction mixture was filtered through a small pad of celite, which was then washed with EtOAc (20 mL). The combined organic phases were then concentrated under reduced pressure to yield a crude red oil. Purification of the crude residue by flash column chromatography (0-4% MeOH/CH_2Cl_2) afforded the desired diol product **X12** (44 mg, 90%) as a red oil.

^{1}H NMR ($CDCl_3$, 500 MHz) δ: 7.64 (1H, s), 7.18 (1H, d, J = 8.6 Hz), 7.09 (1H, s), 6.88 (1H, d, J = 3.9 Hz), 6.77 (1H, dd, J = 8.6, 2.6 Hz), 6.70 (1H, d, J = 2.6 Hz), 6.24 (1H, d, J = 3.9 Hz), 6.13 (1H, s), 5.17 (2H, s),

4.44 (2H, t, *J* = 7.3 Hz), 4.20–4.17 (1H, m), 3.60 (1H, d, *J* = 5.6 Hz), 3.03 (2H, t, *J* = 7.3 Hz), 2.85–2.81 (2H, m), 2.57 (3H, s), 2.42-2.36 (2H, m), 2.29–2.18 (2H, m), 2.25 (3H, s), 1.92–1.81 (3H, m), 1.67–1.63 (1H, m), 1.60-1.54 (1H, m), 1.51–1.32 (4H, m), 0.80 (3H, s). ^{13}C NMR ($CDCl_3$, 125 MHz) δ: 160.6, 156.5, 156.1,144.4, 144.1, 138.0, 135.3, 133.2, 133.0, 128.2, 126.3, 123.9, 122.9, 120.6, 116.7, 114.8, 112.3, 89.9, 78.6, 62.0, 49.9, 47.8, 43.9, 43.8, 38.2, 36.6, 33.6, 29.7, 29.5, 27.2, 25.8, 25.7, 15.0, 12.3, 11.1. IR υ_{max} (film)/cm^{-1} 3371, 2923, 2866, 1602. HRMS (ESI) calcd for $C_{35}H_{42}F_2N_5NaO_3B$ $[M+Na]^+$: *m/z* 651.3277, found *m/z* 651.3262.

II. Standards Preparation

Individual stock solutions of each estrogen were prepared by weighing out the solid material (fine, white crystals) and then dissolving the weighted material in methanol to a concentration of 1 mg/mL. Stock solutions of each BODIPY-tagged estrogen were prepared similarly by weighing out the solid material (fine, red-orange crystals) and then dissolving the weighted material in methanol to a concentration of 1 mg/mL. Individual stock solutions were stored in the dark at -20°C.

On the first day of method evaluation, four sub-stock solutions were prepared by diluting the 1 mg/mL stock solutions in acetonitrile. The first sub-stock consisted of E1 and E2 at 4 μg/mL each, and the second sub-stock contained E3 at 8 μg/mL. The third sub-stock contained BODIPY-E1 and BODIPY-E2 at 9 μg/mL, and the final sub-stock contained BODIPY-E3 also at 9 μg/mL. Sub-stock solutions were stored in the dark at -20°C in amber vials for the total duration of experiments (5 days). The calibration standards and quality controls (QCs) used for system suitability and the method evaluations were prepared from the sub-stock solutions fresh for each batch. The concentrations of each standard and QC are detailed in Table S1.

Table S1: Standards (s#) and quality controls (qcH, qcM, qcL) used for HPLC method evaluation. [a]E3 and BODIPY-E3 are internal standards used for quantifying non-tagged and tagged-estrogens, respectively. [b]The concentrations of s6 were used for the precision and system suitability standard.

	E1		E2		E3[a]		BODIPY-E1		BODIPY-E2		BODIPY-E3[a]	
	ng/mL	μM	ng/mL	μM	ng/mL	μM	ng/mL	μM	ng/mL	μM	ng/mL	μM
s1	60	0.22	60	0.22			45	0.07	45	0.07		
s2	80	0.29	80	0.29			90	0.15	90	0.15		
s3	120	0.44	120	0.44			180	0.29	180	0.29		
s4	160	0.59	160	0.59			270	0.44	270	0.44		
s5	200	0.74	200	0.74	200	0.69	360	0.59	360	0.59	225	0.37
s6[b]	240	0.88	240	0.88			450	0.74	450	0.74		
qcH	220	0.81	220	0.81			405	0.66	405	0.66		
qcM	140	0.52	140	0.52			225	0.37	225	0.37		
qcL	70	0.26	70	0.26			67.5	0.11	67.5	0.11		

III. System Suitability Results

a) b)

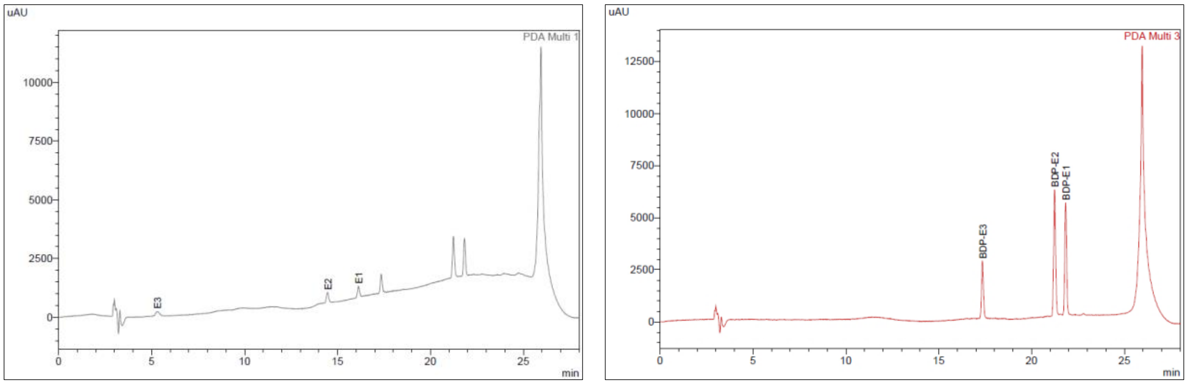

Figure S1. Representative chromatograms of the three methods: E1, E2 and E3 (IS) analyzed by (a) HPLC at 230 nm; (b) BODIPY-E1, BODIPY-E2, and BODIPY-E3 (IS) analyzed by HPLC at 503 nm. The chromatograms show the precision standard at 0.74 μM BODIPY-E1 and BODIPY-E2 and 0.88 μM E1 and E2.

Table S2. System suitability parameters for estrogens and internal standard (IS). [a]Analyte was the first compound eluted.

	Theoretical Plate Number, N		*Resolution, Rs*	
	Untagged	BODIPY-tagged	Untagged	BODIPY-tagged
E1	9.06E+04	2.07E+05	7.4	3.1
E2	5.95E+04	1.78E+05	28.1	20.1
E3 (IS)	2.63E+03	1.30E+05	[a]	5.9

IV. Method Evaluation Calculations and Results

a. Linearity

Linearity for each batch was determined using unweighted linear regression of the concentration (*x*) versus the response (i.e. peak area) ratio of analyte to internal or surrogate standard (*y*). The coefficient of determination (R^2) of the calculated linear regression equation ($y = ax + b$, where a is slope and b is the intercept) was used to evaluate the linearity of the method. Additionally, the percent error (Equation S1, Figure S2) was used to verify the accuracy of the regression equation in accordance with the FDA Bioanalytical Method Validation Guidance for Industry[3]:

$$\%\ \text{Error} = 100\% \times \frac{(x_c - x_i)}{x_i}. \qquad \text{(S1)}$$

Here, x_c represents the concentration as calculated from the standard's response ratio (*y*) using the regression equation and x_i represents the true concentration of the standard.

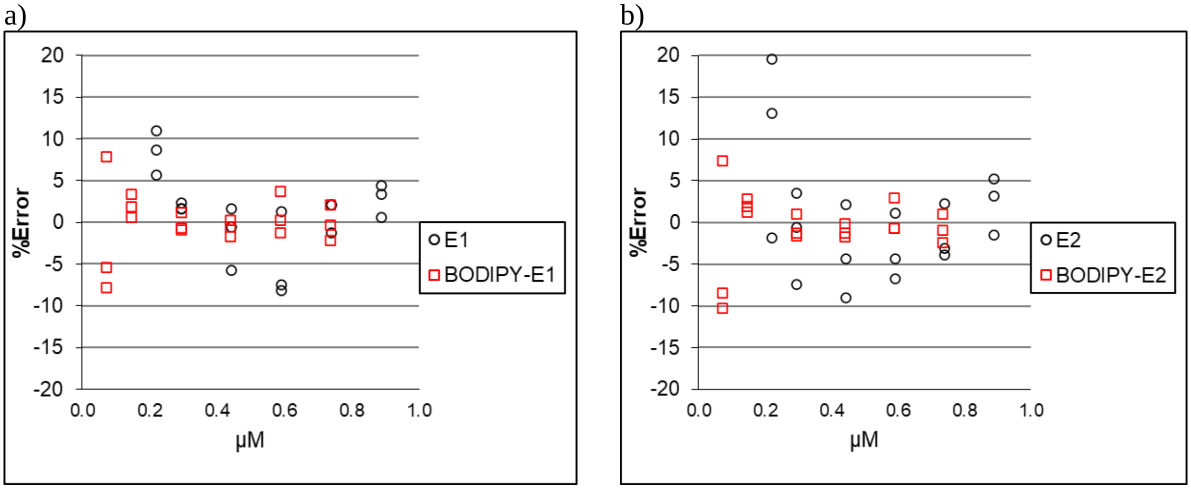

Figure S2: The percent error of each calibration standard for (a) E1 and (b) E2, with and without BODIPY tag. A six-point calibration series was measured as single injections in three separate batches.

b. Precision

Precision was determined by the percent relative standard deviation (%RSD) of a set of replicate injections. Repeatability was evaluated by the percent relative standard deviation (%RSD) for the initial six repeat injections at the start of the first batch (Injections #1-6). Intra-assay precision was evaluated by the %RSD of the initial six repeat injections and final six repeat injections at the end of the first batch (Injections #1-12). Inter-assay precision was evaluated by the %RSD of the repeat injections from the first batch and another six injections at the start of the third, final batch (Injections #1-18).

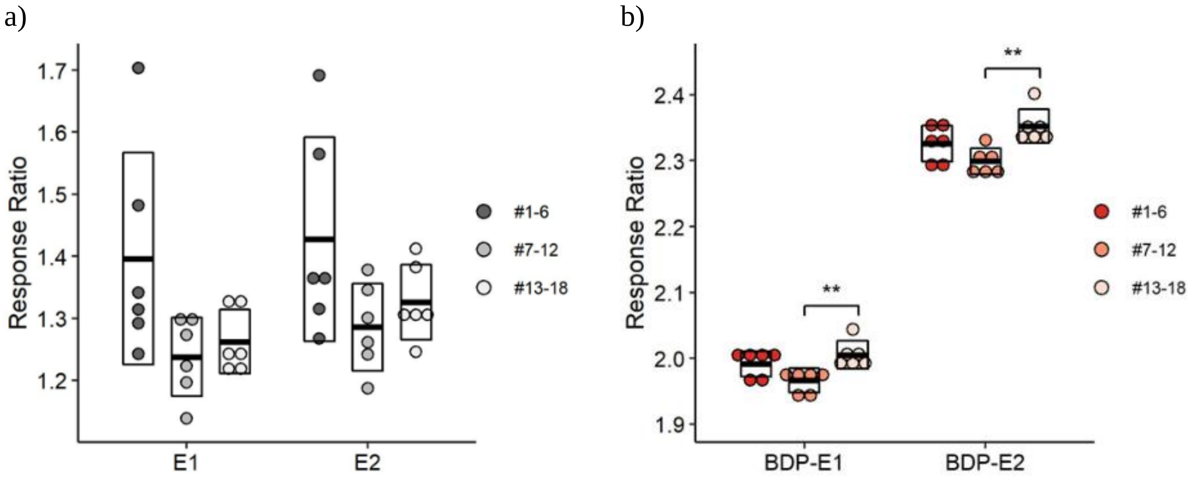

Figure S3: Repeatability and intermediate precision of estrogens (a) E1 and E2 and (b) BODIPY-E1 and BODIPY-E2. Each point represents one repeat injection. The center line of the box represents the mean value of the set of replicate injections (#1-6, #7-12, or #13-18), the upper and lower ends of the box represent standard deviation. One-way ANOVA was used to compare the response ratios of each set of replicates, and the significant variances (P <0.05) are displayed as ** <0.01.

c. Accuracy

Accuracy was determined by the percent error (Equation S1) of the three quality controls measured in duplicate over three separate batches, as recommended by the Bioanalytical Method Validation Guidance for Industry.[3] Here, x_c and x_i are the calculated and true concentrations of the QC sample, respectively. Additionally, the percent recovery (Equation S2) was used as an additional measure of trueness as recommended by the ICH Guidelines and Eurachem Guide to Method Validation:[4,5]

$$\% \text{ Recovery} = 100\% \times \frac{x_c}{x_i}. \qquad \text{(S2)}$$

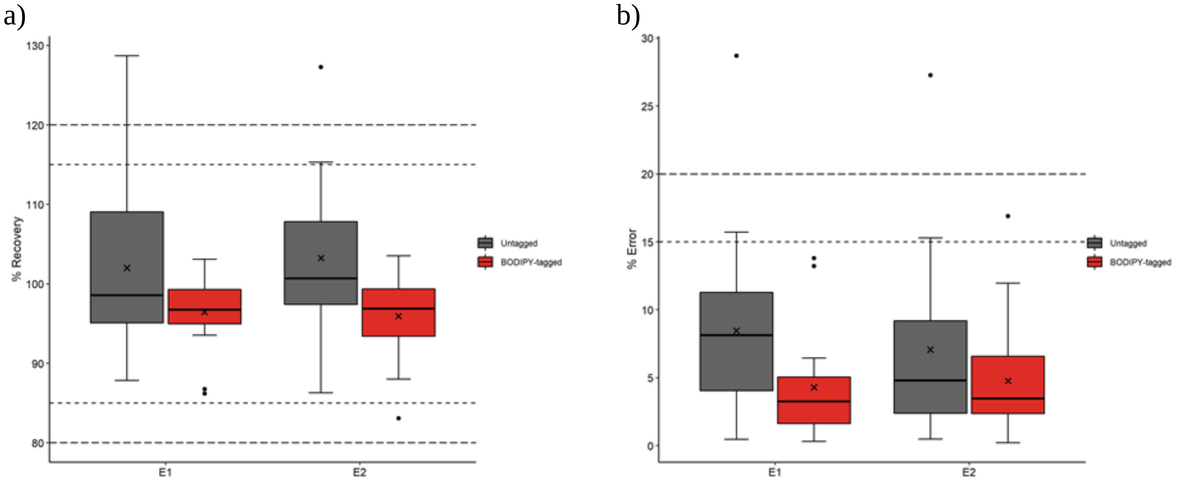

Figure S4: Quantitative accuracy of E1 and E2 with and without BODIPY tag. Each box is the (a) percent recovery or (b) percent error of all three QC levels measured in duplicate in three separate batches (n=18), where the x represents the mean, the center line represents the median value, the upper and lower divisions of the box represent the first and third quartiles, respectively, and the whiskers extend out up to 1.5 times the interquartile range. The short-dashed lines are the acceptance threshold for high and medium QC, and the long-dashed lines are the acceptance threshold for low QC.

A breakdown of the percent error and recovery for each QC level are detailed in Tables S3 for E1 and S4 for E2.

Table S3: The percent recovery and error of the three methods for quantifying E1. The min and max values are the lowest and highest % recovery and % error values determined for the six measurements (i.e. duplicate injections, three batches). The average (Avg) and standard deviation (Stdev) for each level and for all QCs (total) are also reported.

		% Recovery		*% Error*	
QC	Value	Untagged	BODIPY-Tagged	Untagged	BODIPY-Tagged
Low	Min	89.67	86.20	3.89	3.90
	Max	128.70	96.10	28.70	13.80
	Avg	105.68	92.10	10.64	7.90
	Stdev	13.66	4.43	9.46	4.43
Mid	Min	87.84	93.80	2.42	0.32
	Max	109.06	101.36	12.16	6.20
	Avg	97.62	98.38	8.40	2.07
	Stdev	9.45	2.56	3.36	2.14
High	Min	94.96	95.20	0.46	0.64
	Max	115.71	103.11	15.71	4.80
	Avg	102.73	98.94	6.35	2.93
	Stdev	8.66	3.28	5.96	1.36
**TOTAL**	**Avg**	**102.01**	**96.47**	**8.46**	**4.30**
	**Stdev**	**10.72**	**4.59**	**6.58**	**3.83**

Table S4: The percent recovery and error of the three methods for quantifying E2. The min and max values are the lowest and highest % recovery and % error values determined for the six measurements (i.e. duplicate injections, three batches). The average (Avg) and standard deviation (Stdev) for each level and for all QCs (total) are also reported.

		% Recovery		*% Error*	
QC	Value	Untagged	BODIPY-Tagged	Untagged	BODIPY-Tagged
Low	Min	94.41	83.11	0.49	2.34
	Max	127.27	97.66	27.27	16.89
	Avg	107.94	91.38	9.80	8.62
	Stdev	11.81	5.10	9.99	5.10
Mid	Min	86.29	92.67	0.88	0.21
	Max	109.66	100.21	13.71	7.33
	Avg	100.14	97.76	5.95	2.31
	Stdev	8.36	2.77	5.24	2.70
High	Min	95.98	94.62	1.33	0.86
	Max	113.93	103.54	13.93	5.38
	Avg	101.68	98.65	5.45	3.39
	Stdev	7.33	3.75	4.64	1.58
**TOTAL**	**Avg**	**103.25**	**95.93**	**7.07**	**4.77**
	**Stdev**	**9.46**	**5.01**	**6.91**	**4.31**

d. Instrument Limits of Detection and Quantitation

The instrument limits of detection (LOD) and quantitation (LOQ) were calculated for each calibration curve using Equation S3,

$$\text{Instrument Limit} = k\frac{s_b}{S} \tag{S3}$$

where k is 3.3 for LOD and 10 for LOQ, s_b is the standard error of the intercept, and S is the regression slope. This model is based on the ICH Guidelines for determining limits of detection and quantitation, which states that the standard deviation of the y-intercept may be used as representation for standard deviation of the response.[4] Because the estimates of the instrumental limits of detection and quantitation are based on the regression curves, these values were also determined for each individual batch.

V. LLE Recovery Results

Table S5: Percent recovery values for the different LLE methods assessed. Recovery values are the mean (± standard deviation) of 3 replicate samples extracted (n=3) at different concentrations. The "Mean" concentration is the average recovery across all concentrations assessed (**bold** text). N.D. – Not detected.

					% Recovery			
Version	Conc.	E1	E2	E3	BODIPY-E1	BODIPY-E2	BODIPY-E3	BODIPY-N3
Diluted + Filtered	Low	71.0 ± 3.8	69.7 ± 4.6	98.3 ± 40.4	66.4 ± 1.1	64.9 ± 1.5	64.3 ± 2.1	65.5 ± 2.1
	Mid	70.1 ± 2.8	67.9 ± 2.0	62.7 ± 6.1	65.7 ± 1.4	65.3 ± 1.5	64.1 ± 1.3	64.5 ± 0.9
	High	73.4 ± 0.8	71.3 ± 1.1	67.8 ± 2.7	71.8 ± 1.1	72.2 ± 0.8	70.8 ± 0.9	69.2 ± 0.9
	**Mean**	**71.5 ± 2.8**	**69.6 ± 2.9**	**76.3 ± 26.4**	**67.9 ± 3.1**	**67.5 ± 3.7**	**66.4 ± 3.6**	**66.4 ± 2.5**
HPβ-CDX + Filtered	Mid	90.0 ± 3.8	100.9 ± 21.4	N.D.	43.2 ± 4.4	66.6 ± 2.5	81.6 ± 1.9	66.3 ± 1.5
	High	75.7 ± 12.7	61.6 ± 11.7	N.D.	26.6 ± 6.8	44.7 ± 10.5	65.1 ± 14.0	52.9 ± 10.3
	**Mean**	**82.9 ± 11.5**	**81.3 ± 26.5**	**N.D.**	**34.9 ± 10.4**	**55.7 ± 13.8**	**73.4 ± 12.7**	**59.6 ± 9.9**
HPβ-CDX + Diluted + Filtered	Low	71.8 ± 5.1	80.6 ± 6.2	N.D.	68.0 ± 2.8	69.9 ± 2.6	71.3 ± 2.5	71.6 ± 2.8
	Mid	69.7 ± 0.8	65.8 ± 1.4	15.6 ± 1.2	65.7 ± 0.7	66.1 ± 0.9	67.0 ± 0.9	68.0 ± 0.5
	High	65.3 ± 1.3	63.5 ± 2.1	34.1 ± 1.7	61.9 ± 2.6	62.2 ± 2.9	62.8 ± 3.3	63.4 ± 3.4
	**Mean**	**69.0 ± 3.9**	**70.0 ± 8.7**	**16.6 ± 14.8**	**65.2 ± 3.3**	**66.0 ± 3.9**	**67.0 ± 4.3**	**67.7 ± 4.2**

VI. BODIPY Estrogens NMR and HRMS Spectra

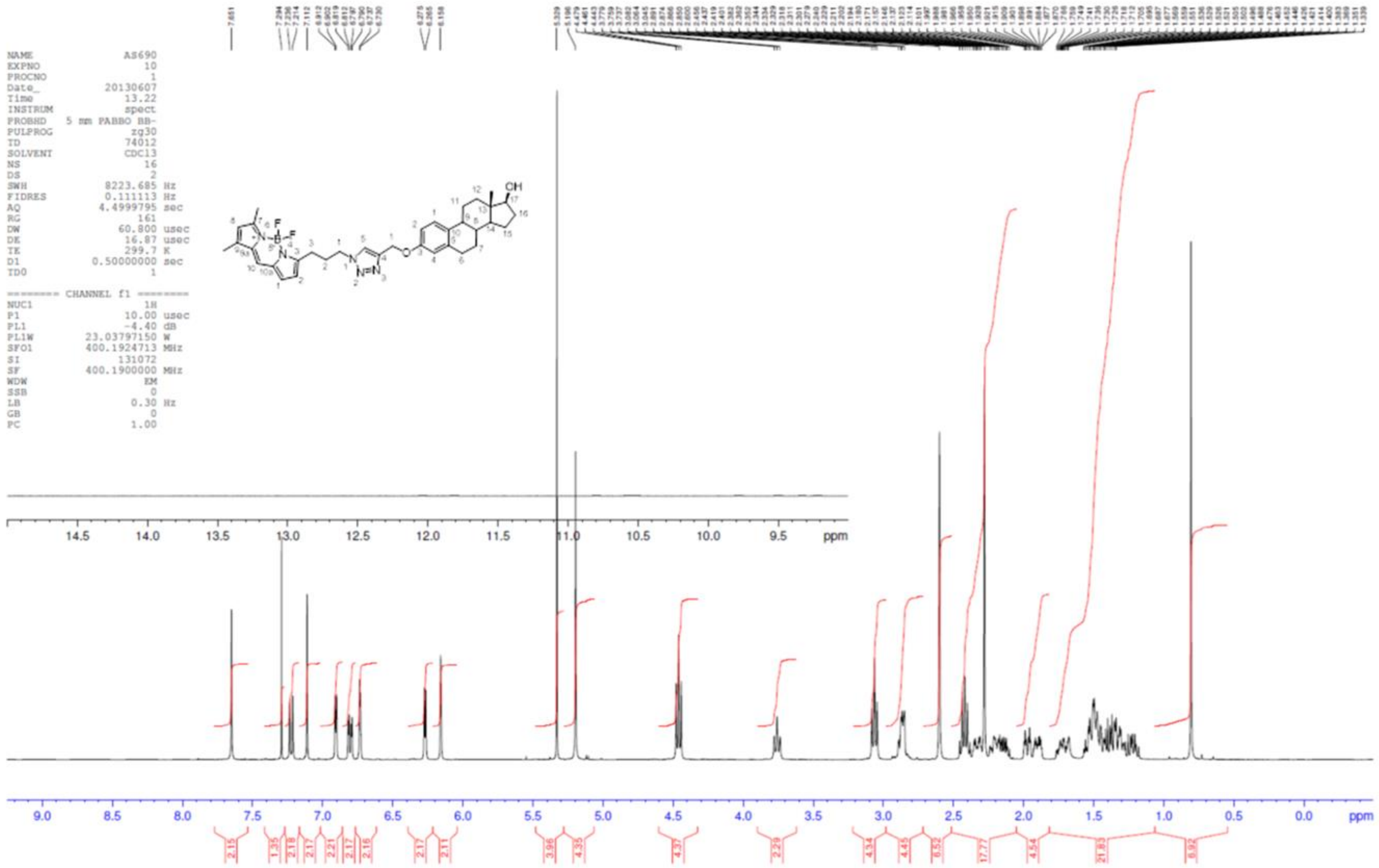

Figure S5: ^{1}H NMR spectrum for BODIPY-estradiol (**X10**)

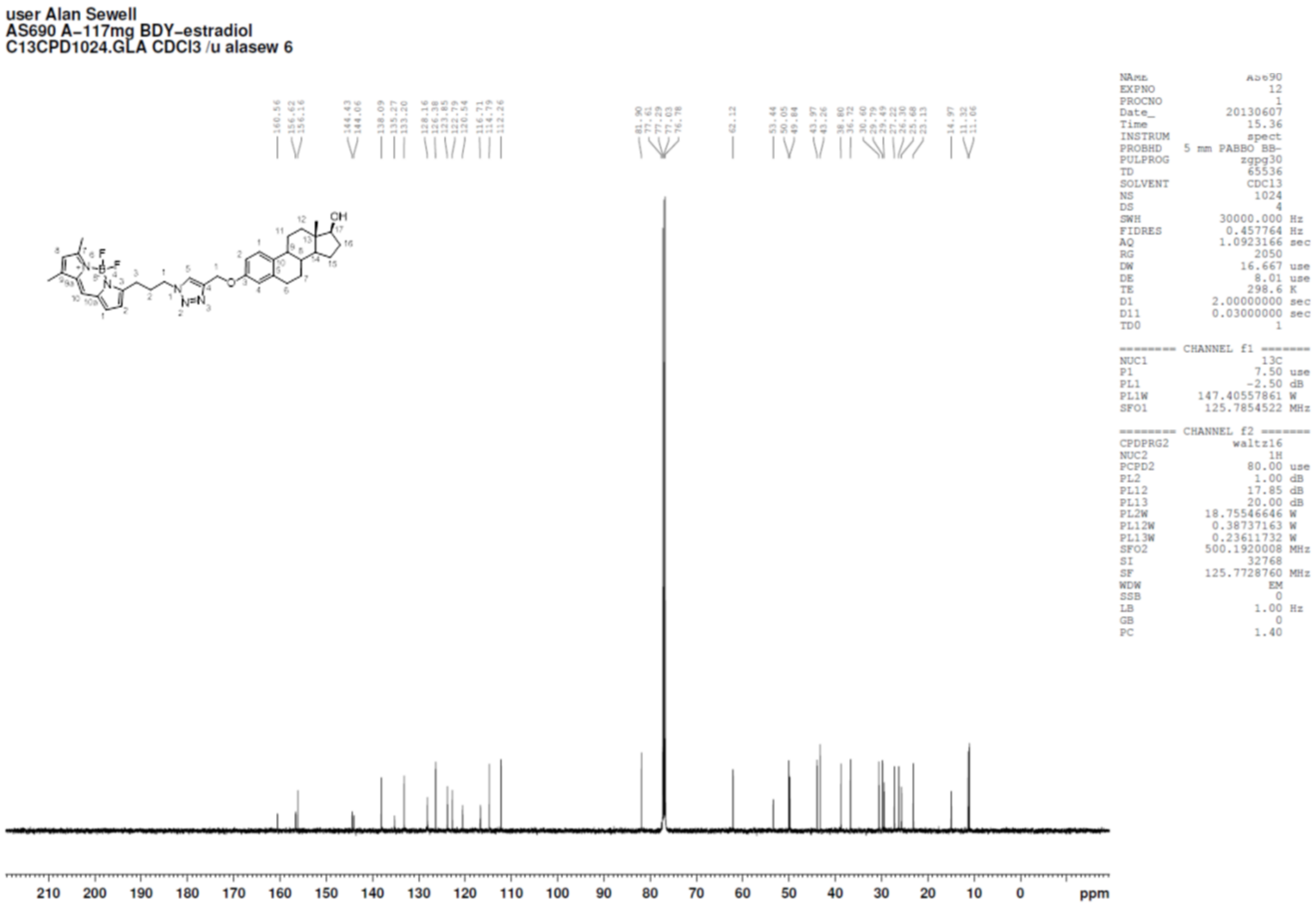

Figure S6: ^{13}C NMR spectrum for BODIPY-estradiol, 1 (**X10**).

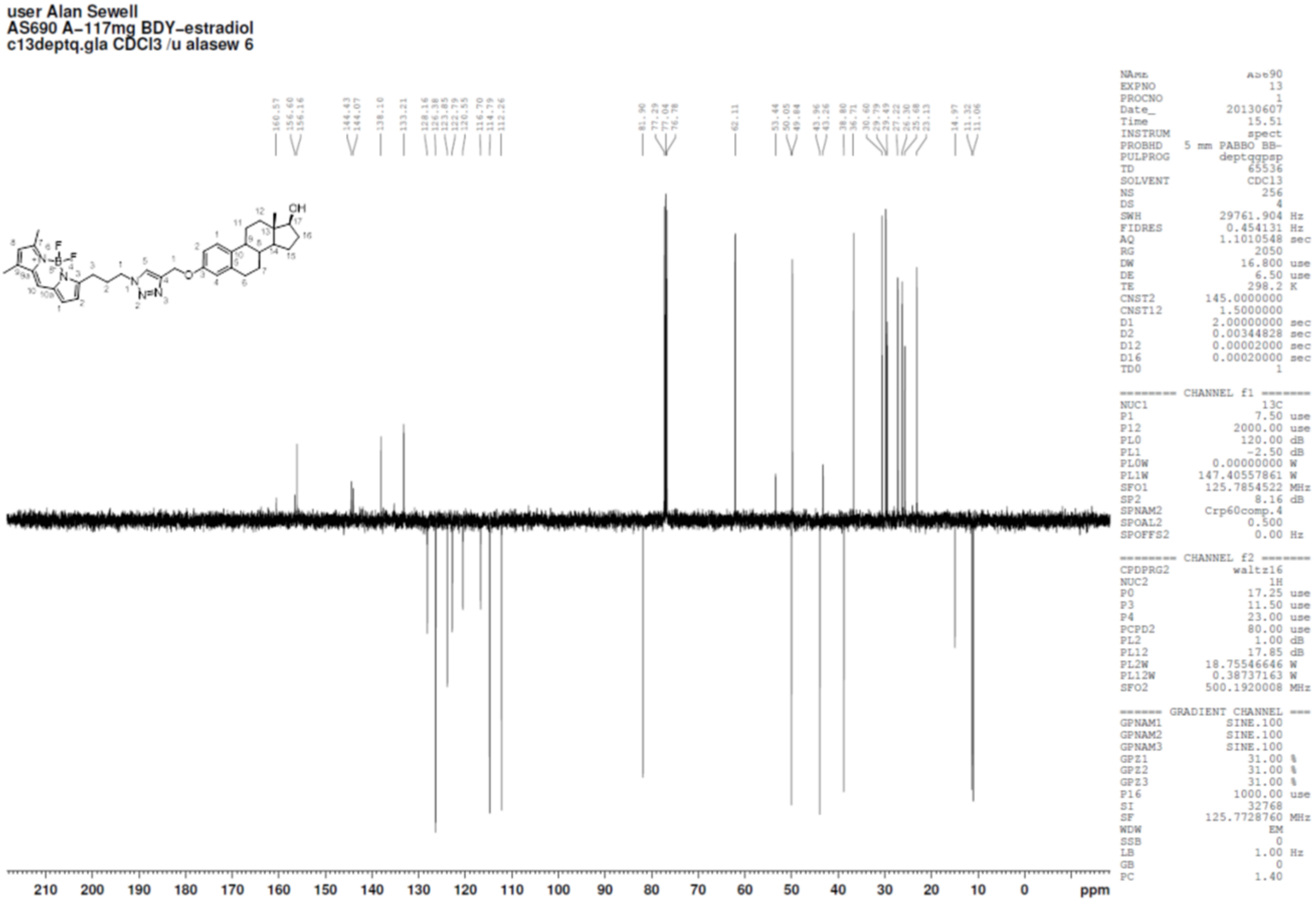

Figure S7: ^{13}C NMR spectrum for BODIPY-estradiol, 2 (**X10**).

BODIPY - ESTRADIOL
AS-690

Mass Spectrum SmartFormula Report

**Analysis Info**			
Analysis Name	D:\Data\training\55469-000002.d	Acquisition Date	7/2/2013 10:07:45 AM
Method	SM MS 50 to 800.m	Operator	user
Sample Name	SEWELL AS690	Instrument / Ser#	micrOTOF-Q 74
Comment			

**Acquisition Parameter**			
Source Type	ESI	Ion Polarity	Positive
Scan Begin	50 m/z		
Scan End	800 m/z		

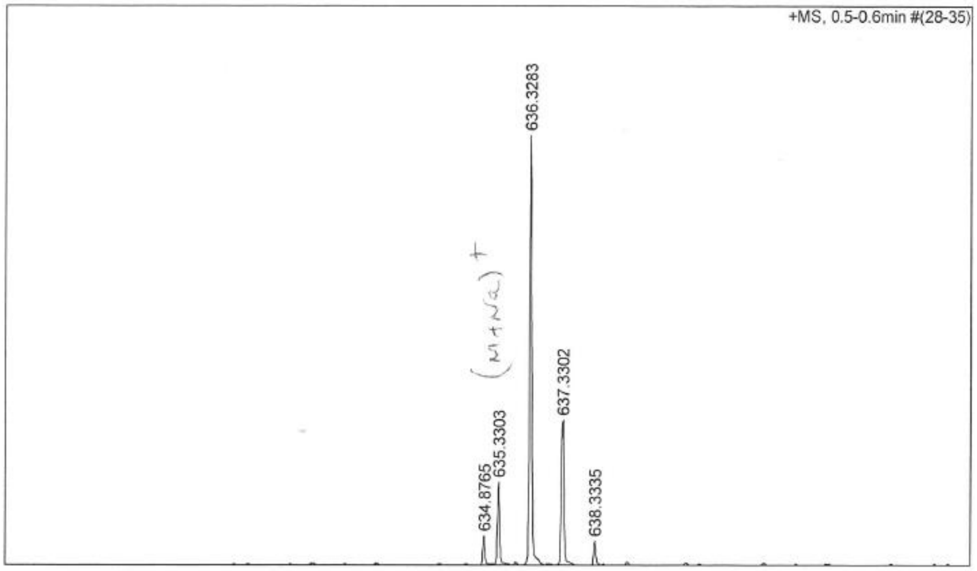

Formula	z	m/z	Meas. m/z	err [ppm]	err [mDa]
✓ C 35 H 42 F 2 N 5 Na O 2 ^10B	1+	635.3328	635.3303	4.0	2.5
C 23 H 170 F 2 N 5 Na O 3 ^10B	1+	636.3293	636.3283	1.7	1.1

Bruker Compass DataAnalysis 4.0 printed: 7/2/2013 10:13:12 AM Page 1 of 2

Figure S8: HRMS spectrum for BODIPY-estradiol (**X10**).

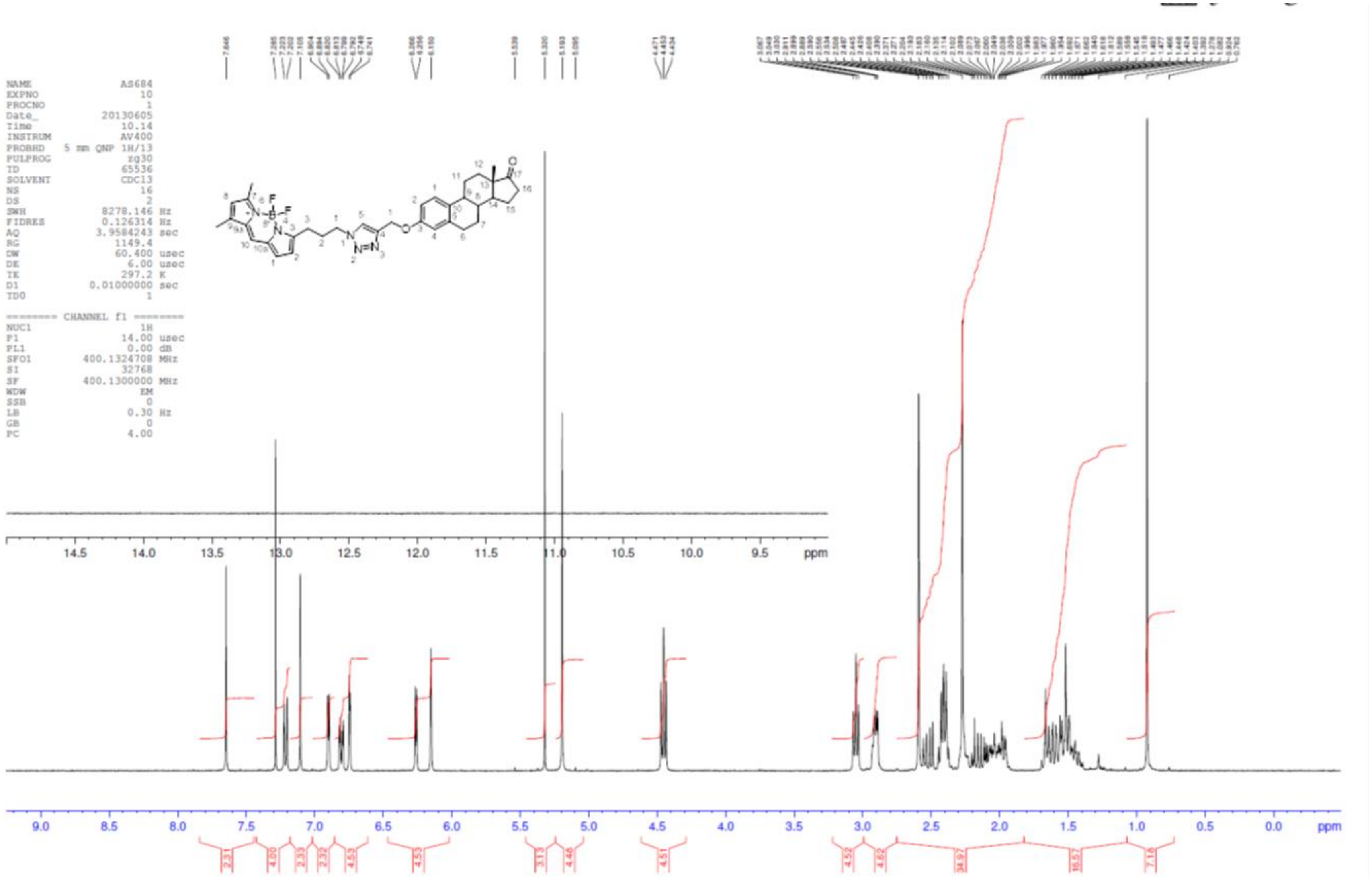

Figure S9: ^{1}H NMR spectrum for BODIPY-estrone (**X9**).

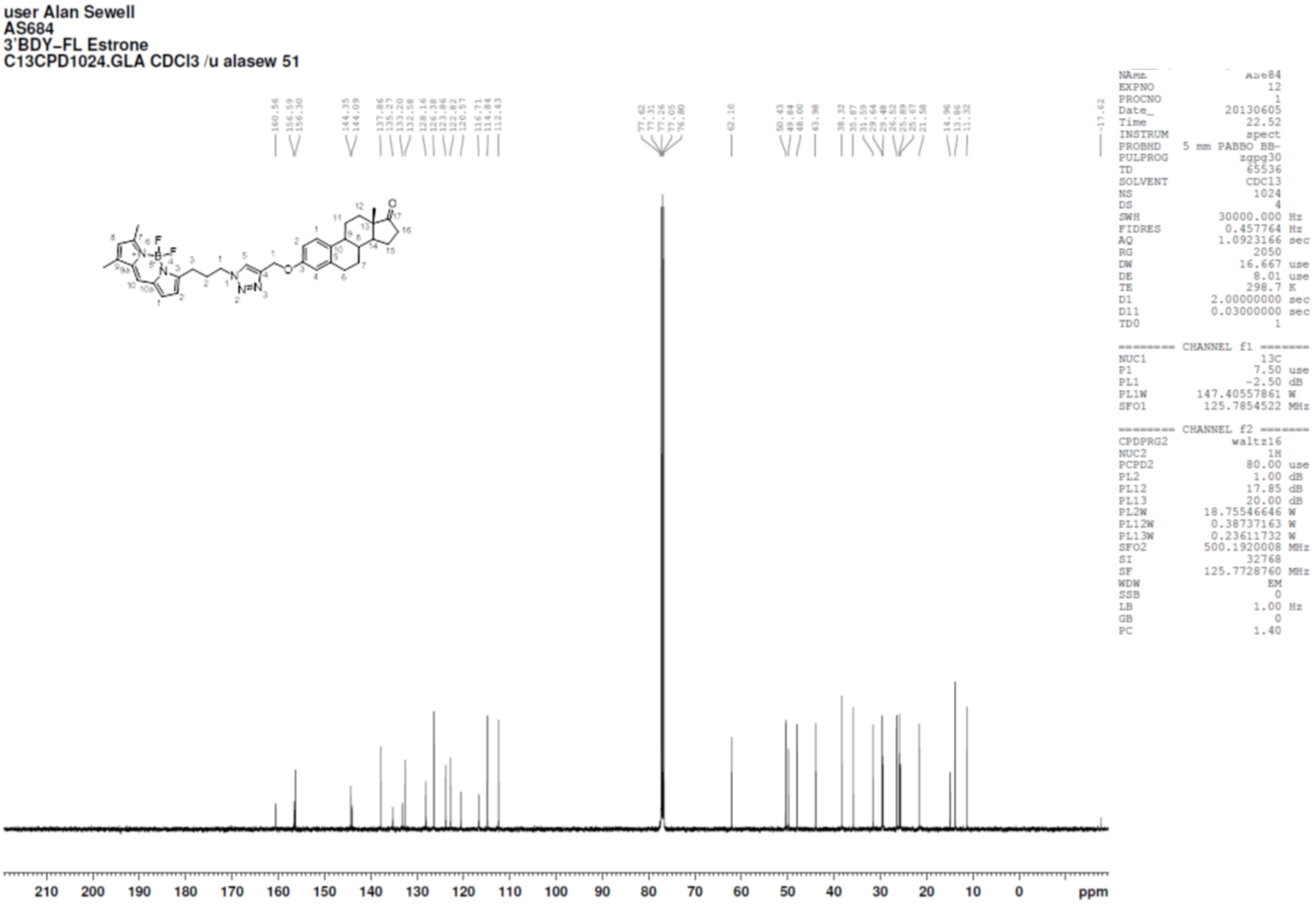

Figure S10: ^{13}C NMR spectrum for BODIPY-estrone, 1 (**X9**).

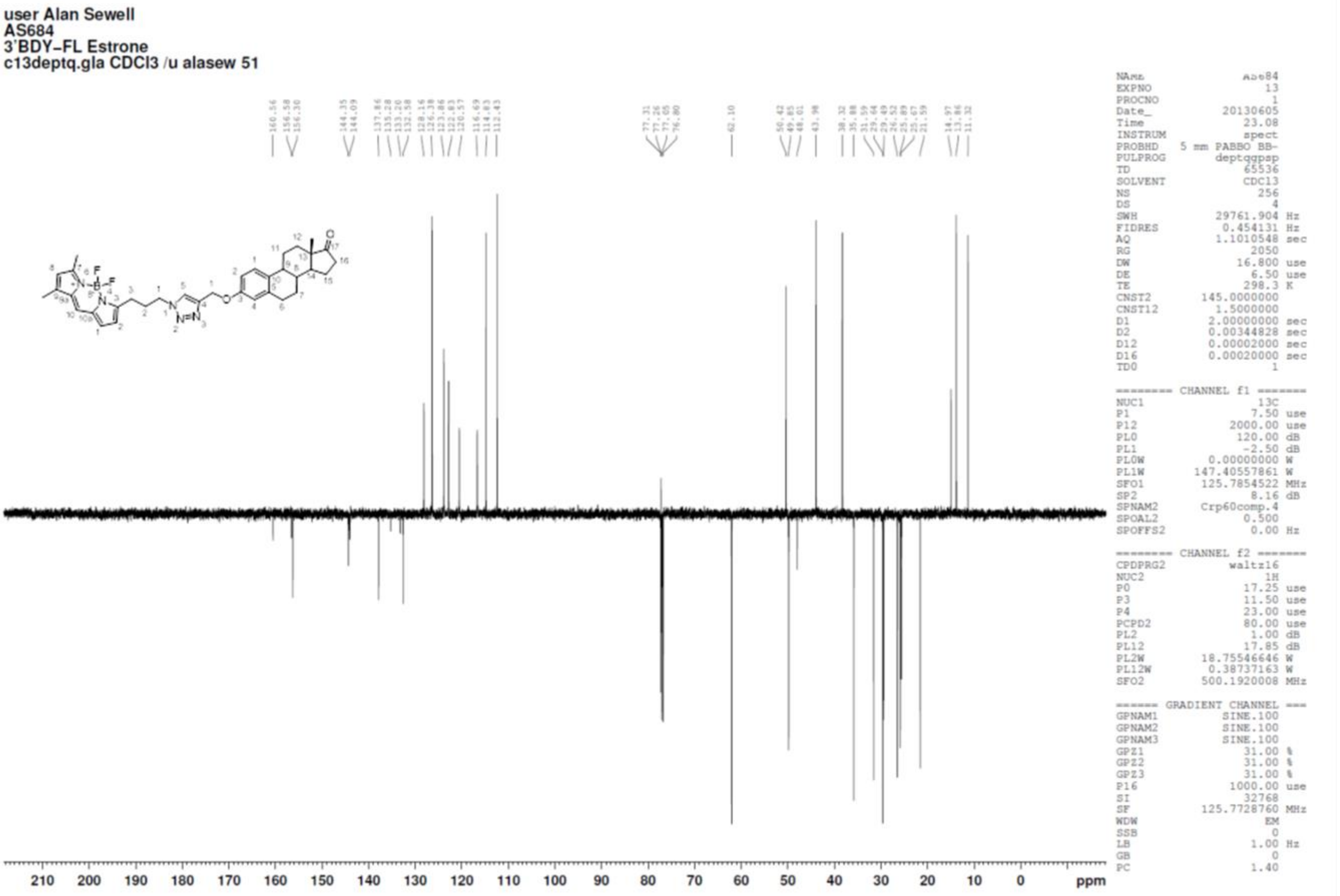

Figure S11: ^{13}C NMR spectrum for BODIPY-estrone, 2 (**X9**).

BODIPY-ESTRONE
AS-684

Mass Spectrum SmartFormula Report

Analysis Info

Analysis Name	D:\Data\training\55468-000002.d	Acquisition Date	7/2/2013 10:18:58 AM
Method	SM MS 50 to 800.m	Operator	user
Sample Name	SEWELL AS684	Instrument / Ser#	micrOTOF-Q 74
Comment			

Acquisition Parameter

Source Type	ESI	Ion Polarity	Positive
Scan Begin	50 m/z		
Scan End	800 m/z		

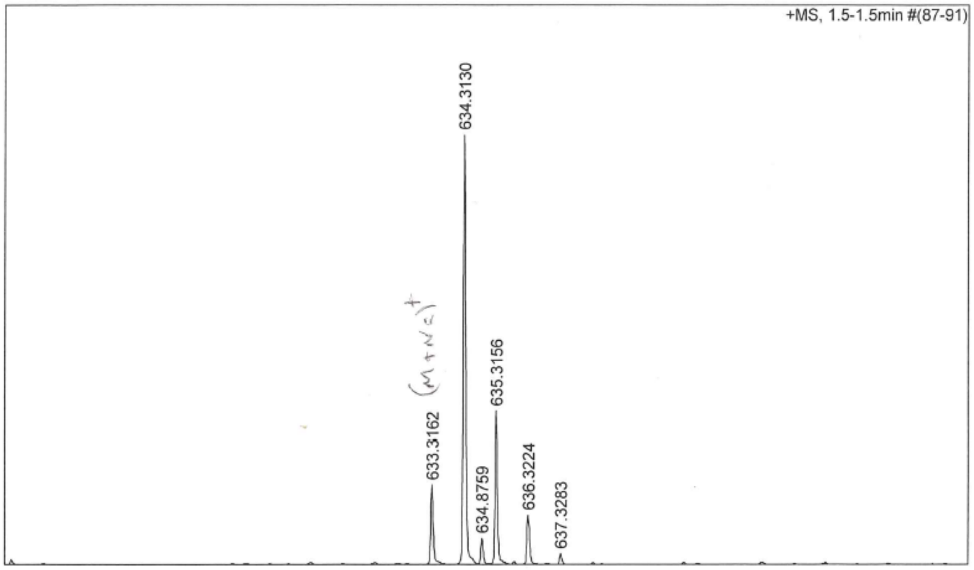

Formula	z	m/z	Meas. m/z	err [ppm]	err [mDa]
	1+		633.3162		
C 35 H 40 F 2 N 5 Na O 2 ^10B		633.3172		1.5	0.9

Bruker Compass DataAnalysis 4.0 printed: 7/2/2013 10:23:55 AM Page 1 of 2

Figure S12: HRMS spectrum for BODIPY-estrone (**X9**).

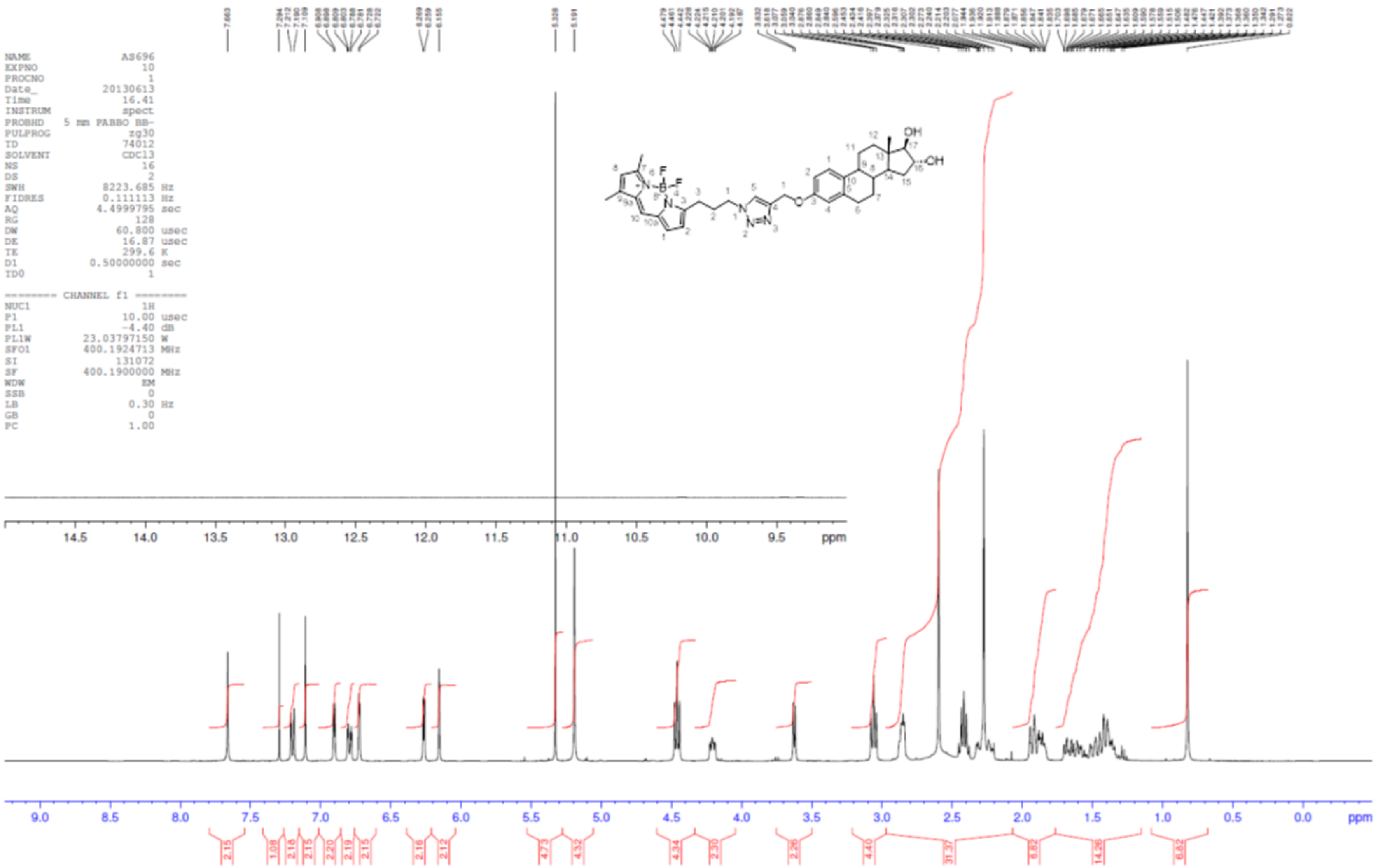

Figure S13: ^{1}H NMR spectrum for BODIPY-estriol (**X12**).

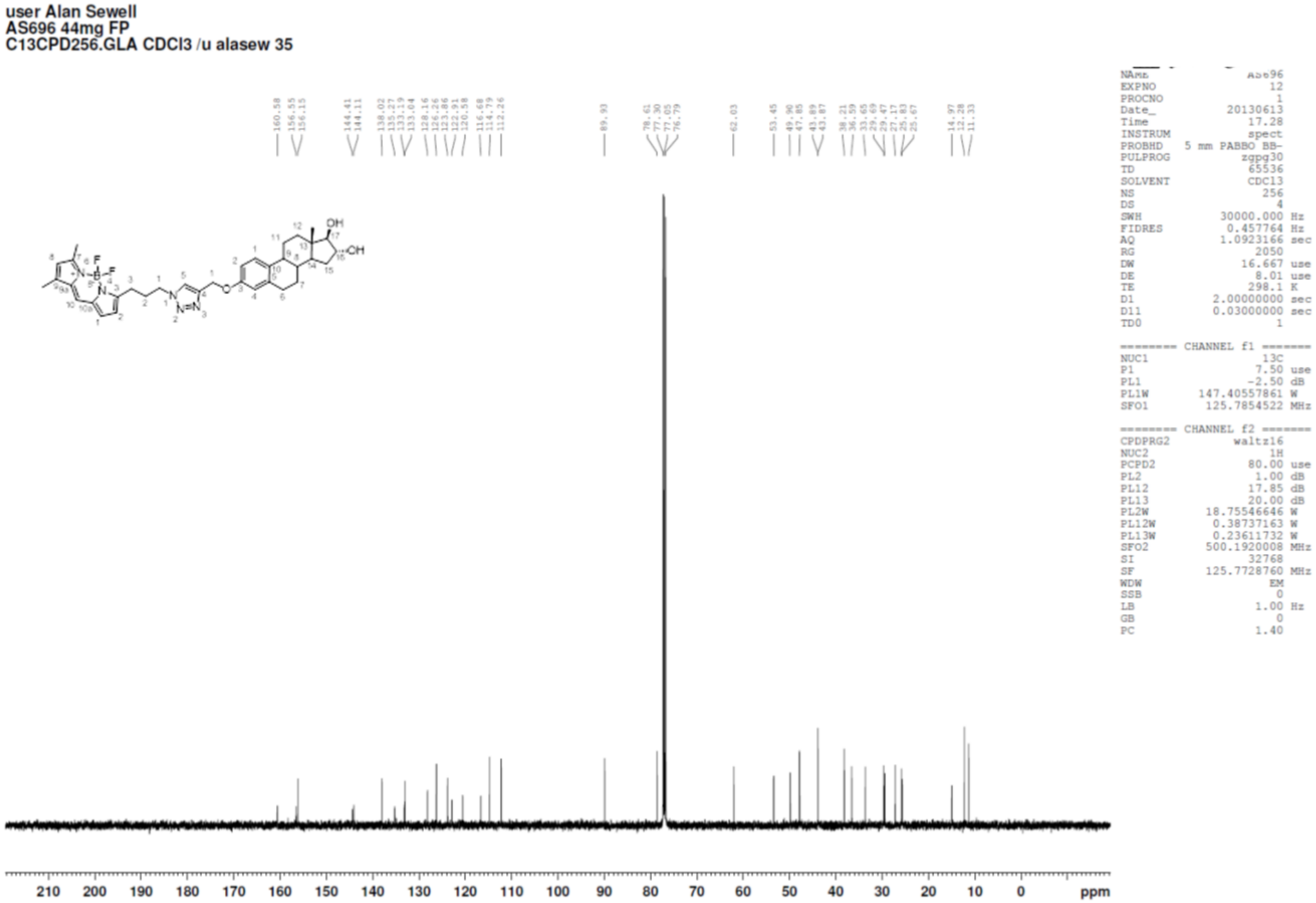

Figure S14: ^{13}C NMR spectrum for BODIPY-estriol, 1 (**X12**).

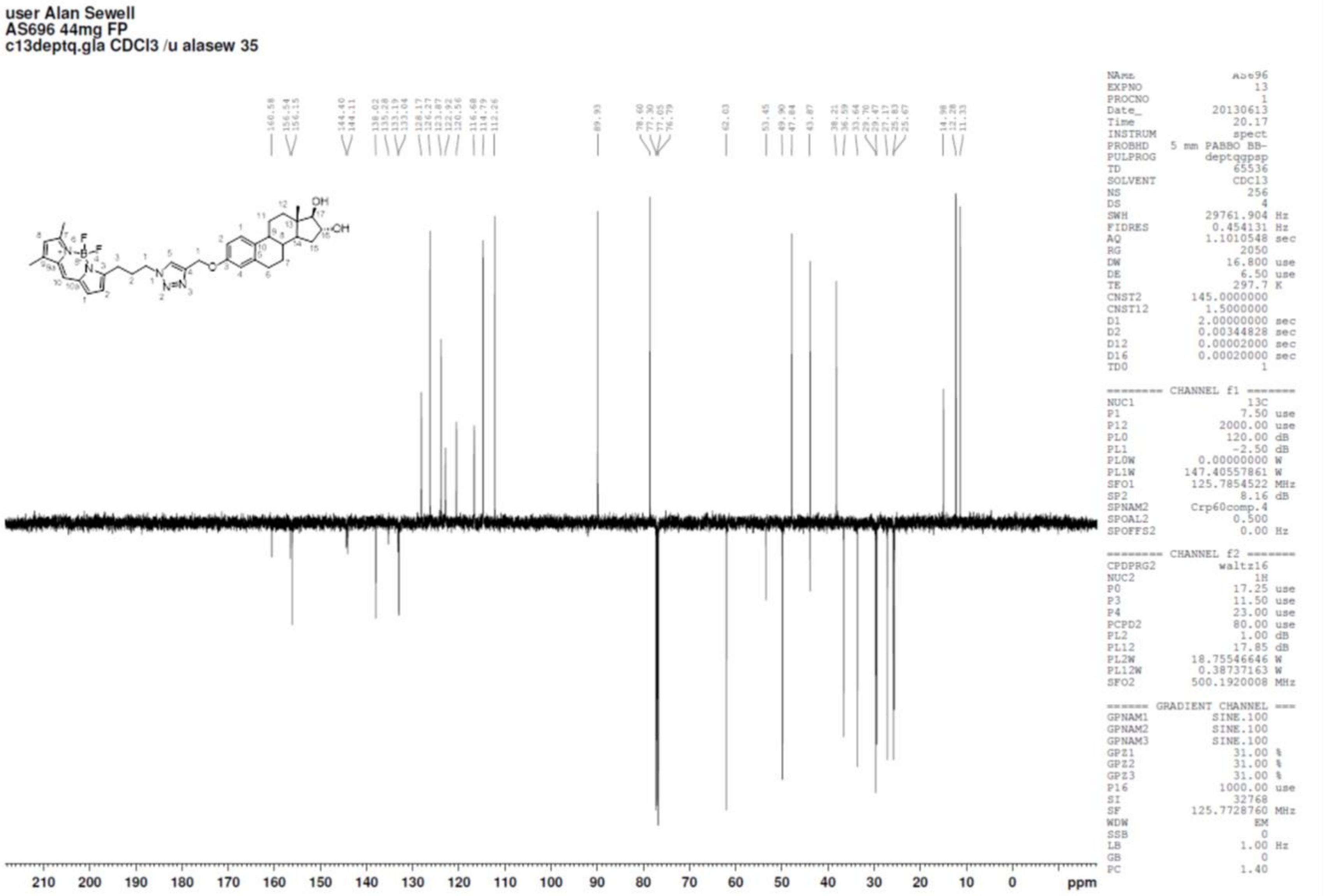

Figure S15: ^{13}C NMR spectrum for BODIPY-estriol, 2 (**X12**).

BODIPY - ESTRIOL
AS69C

Mass Spectrum SmartFormula Report

Analysis Info

Analysis Name	D:\Data\training\55471-000002.d	Acquisition Date	7/2/2013 9:41:43 AM
Method	SM MS 50 to 800.m	Cperator	user
Sample Name	SEWELL AS696	Instrument / Ser#	micrOTOF-Q 74
Comment			

Acquisition Parameter

Source Type	ESI	Ion Polarity	Positive
Scan Begin	50 m/z		
Scan End	800 m/z		

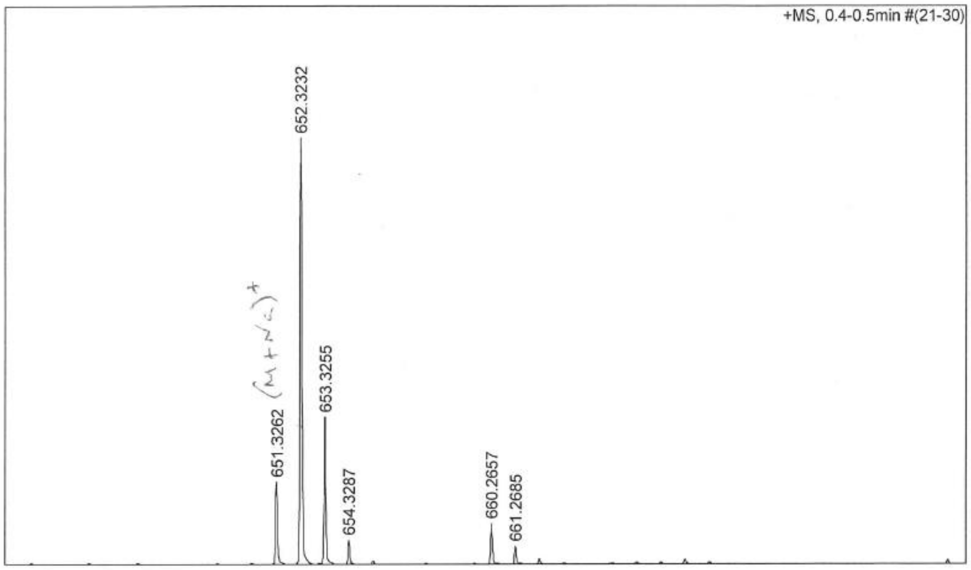

Formula	z	m/z	Meas. m/z	err [ppm]	err [mDa]
	~~1+~~		~~561.3262~~		
	1+		651.3262		
C 35 H 42 F 2 N 5 Na O 3 ^10B		651.3277		2.4	1.6

Bruker Compass DataAnalysis 4.0 printed: 7/2/2013 9:47:12 AM Page 1 of 1

Figure S16: HRMS spectrum for BODIPY-estriol (**X12**).

VII. References

(1) Hansen, A. M.; Sewell, A. L.; Pedersen, R. H.; Long, D.-L.; Gadegaard, N.; Marquez, R. Tunable BODIPY Derivatives Amenable to 'Click' and Peptide Chemistry. *Tetrahedron* **2013**, *69* (39), 8527–8533. https://doi.org/10.1016/j.tet.2013.05.037.

(2) Ramírez-López, P.; de la Torre, M. C.; Montenegro, H. E.; Asenjo, M.; Sierra, M. A. A Straightforward Synthesis of Tetrameric Estrone-Based Macrocycles. *Org. Lett.* **2008**, *10* (16), 3555–3558. https://doi.org/10.1021/ol801313g.

(3) U.S. Department of Health and Human Services; Food and Drug Administration; Center for Drug Evaluation and Research (CDER); Center for Veterinary Medicine (CVM). Bioanalytical Method Validation Guidance for Industry, 2018.

(4) Abraham, J. International Conference On Harmonisation Of Technical Requirements For Registration Of Pharmaceuticals For Human Use. In *Handbook of Transnational Economic Governance Regimes*; Brouder, A., Tietje, C., Eds.; Brill, 2009; pp 1041–1054. https://doi.org/10.1163/ej.9789004163300.i-1081.897.

(5) *The Fitness for Purpose of Analytical Methods: A Laboratory Guide to Method Validation and Related Topics*; 2014.
